# Zinc metal complexes synthesized by a green method as a new approach to alter the structural and optical characteristics of PVA: new field for polymer composite fabrication with controlled optical band gap

**DOI:** 10.1039/d4ra04228j

**Published:** 2024-08-20

**Authors:** Dana S. Muhammad, Dara M. Aziz, Shujahadeen B. Aziz

**Affiliations:** a Department of Physics, College of Education, University of Sulaimani Old Campus, Kurdistan Regional Government Sulaimani 46001 Iraq; b Department of Chemistry, College of Science, University of Raparin Kurdistan Region Ranya 46012 Iraq; c Research and Development Center, University of Sulaimani Qlyasan Street, Kurdistan Regional Government Sulaymaniyah 46001 Iraq shujahadeenaziz@gmail.com; d Department of Physics, College of Science, Charmo University Chamchamal 46023 Sulaymaniyah Iraq

## Abstract

The current study employed a novel approach to design polymer composites with modified structural and declined optical band gaps. The results obtained in the present work for polymer composites can be considered an original method to make a new field for research based on green chemistry. Natural dyes extracted from green tea were mixed with hydrated zinc acetate (Zn(CH_3_COO)_2_·2H_2_O) to produce a metal complex. FTIR results comprehensively established the formation of the Zn-metal complex. The interaction among various components of PVA : Zn-metal complex composite was investigated using FTIR spectroscopy. The non-existence of anion bands of acetate in the Zn-metal complex spectrum confirms the formation of the Zn-metal complex. XRD analysis reveals that the Zn-metal complex improves the amorphous phase of the PVA-based composites. The absorption edge of the doped films shifted towards the lower photon energies. Optical dielectric properties were used to determine *N*/m*, *ε*_∞_, *τ*, *μ*_opt_, *ω*_p_, and *ρ*_opt_; the W–D model was used to calculate *E*_d_, *E*_o_ and *n*_o_ parameters. The optical dielectric loss parameter was used to determine the optical band gap while the Tauc model was employed to identify various types of electron transitions. The optical energy band gap was 6.05 eV for clean PVA while it decreased to 1 eV for PVA inserted with the Zn-metal complex. The increase in Urbach energy from 0.26 eV to 0.45 eV is an evidence of the boost of amorphous phases in PVA : Zn-metal complex composites. The nonlinear refractive index and the first-order and second-order nonlinear optical susceptibilities were determined. The value of *E*_o_ obtained from the W–D model closely matches the optical energy band gap obtained from the Tauc model, which indicates the precision of the analysis in the present study. The increase in SELF and VELF in the composite films establishes that new energy states assigned to the added Zn-metal complex amplify the probability of light–matter interaction.

## Abstract 2

The current study employed a novel approach to design polymer composites with modified structural and declined optical band gaps.

## Graphical abstract

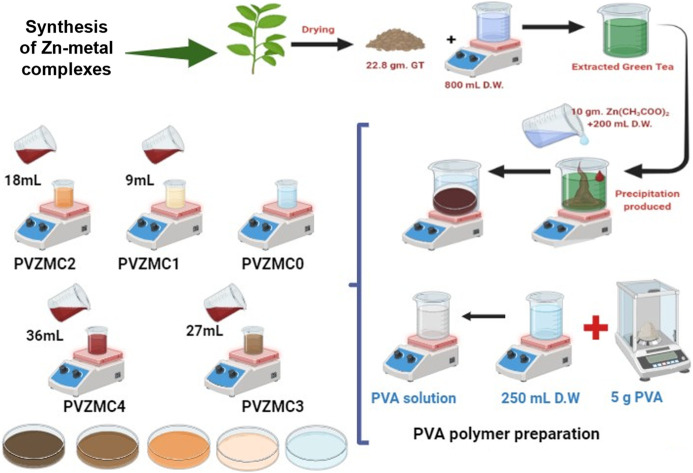

## 1. Introduction

Recently, research interests in polar organic polymers such as polyvinyl alcohol (PVA) have been in progress due to their strong chemical and thermal stabilities, affordability, mechanical flexibility, high biocompatibility, and transparency. Because PVA chains are hydrogen bonded, they are a synthetic semicrystalline structured polymer.^1–4^ The most pertinent feature of PVA is that, in addition to being non-toxic, it is also a readily degradable and water-soluble crystalline polymer.^5,6^ PVA is a biodegradable substance used in paper coating and textile sizing. This particular polymer is typically employed in the pharmaceutical and chemical industries after being blended with other polymers.^7,8^ Additionally, PVA possesses hydrophilic properties, the ability to form films, and a high density of reactive chemical functional groups. These functional (OH) groups facilitate the cross-linking of PVA with doping materials.^9^

Wastewater has emerged as a significant source of pollution and a serious threat to human health in recent years.^10^ Water pollution caused by persistent, hazardous, and bio-accumulative organic and inorganic contaminants has been linked to several environmental issues.^11^ Consequently, using natural materials made from renewable resources is one of the strategies used to address these challenges. It concerns the advancement of green chemistry, which is centred on employing biopolymers and natural dyes.^12^ Dye-doped polymers have gained popularity due to their numerous advantages. Their versatility makes them useful in photonic devices, both linear and nonlinear.^13^ Novel optical telecommunication devices, cutting-edge nanoscale lasers, and chip-integrated photonic biosensors all use dye-doped polymers.^14^ Electronic sensors, optical fibers, solar cells, optophotonic devices, and dye-doped polymeric materials are some of the promising new uses of these materials.^15^ Optical characterization of polymer films provides a wealth of significant physical data (refractive index *n*, extinction coefficient *k*, optical energy gap *E*_g_, and dielectric constant *ε*) that are critical for various applications.^16^

Examining the absorption edge and refractive index can help us understand the type of transitions that occurs between the highest occupied molecular orbital (HOMO) and the lowest unoccupied molecular orbital (LUMO).^17^ To our understanding, metal complexes can be inserted into polymers to create polymer composite films with the desired optical properties. Researchers have made great strides in the field of organic-inorganic hybrid materials over the past 20 years; this is an intriguing and potentially pivotal area of study. The optical characteristics of PVA mixed with metal complexes have been studied in the literature. Transition metal complexes are a varied group of compounds that encompasses bioinorganic, Werner, and organometallic complexes. They have significant uses in various fields, such as catalysis, nanomaterials (such as electronic devices), medicinal chemistry, and renewable energies.^18,19^ Because of their high surface-to-bulk ratio, inorganic particles inserted into the host polymer may cause considerable changes in the host's properties.^20–22^ Previous studies employed many methods for the production of ligands to produce metal complexes, such as (C_15_H_10_N_4_O_7_SCl),^23^ 4-[(2-amino-4-phenylazo)-methyl]*N*-[(benzoyl amino)-thioxo methyl]-cyclohexane carboxylic acid,^24^*N*-[(benzoyl amino)-thioxo methyl], the benzoyl isothiocyanate–proline reaction,^25^ and 4-[(2-amino-4-phenylazo)-methyl]-cyclohexane carboxylic acid.^26^ According to Samuel *et al.*, there are several issues with the ligand method for synthesizing metal complexes using organic solvents under reflux. These issues encompass excessive use of organic solvents, prolonged reaction times, environmentally harmful solvents, reduced product yields, and harsh reflux conditions.^27^ In the present study, we offer a new technique for preparing metal complexes, namely the synthesis of Zinc metal complex (Zn-metal complex) using green tea (GT) dye and Zn(CH_3_COO)_2_ salt. The GT dye is renowned for its significant phytochemical components in metallic ion reduction.^28,29^ The composition of fresh, young green tea leaves when analyzed for dry weight is generally as follows: Polyphenols comprise 20–35% of the composition, while cellulose, lignin, starch, and other substances account for 20–30%. Proteins make up 10–20%, fats make up 3–9%, mineral substances make up 4–8%, polysaccharides make up 4–7%, amino acids make up 3–4%, caffeine makes up 2–4%, chlorophyll and carotenoids make up 2–3%, and volatile chemicals are also present.^30,31^ The polyphenolic level in green tea types varies depending on factors such as the harvesting season, cultivars, cultivation circumstances, and manufacturing procedure. The main catechins found in tea leaves include (–)-epigallocatechin gallate (EGCG), (–)-epi-gallocatechin (ECC), (–)-epicatechin gallate (ECG), and (–)-epicatechin (EC);^20,32^ it has also been demonstrated that dyes derived from tea, specifically black tea (BT), contain sufficient ligands with functional groups, such as OH, NH, NH_2_, and C=O. Based on Aziz's approach, tea dyes are an environmentally friendly approach to producing Sn^2+^-PPH metal–organic frameworks.

According to previous studies, several approaches have been carried out to modify the optical properties of PVA, such as the insertion of nanofillers, polymer blending, ceramic filler, and salt addition. It was found that nano-Ag insertion into PVA decreased the optical band gap from 4.92 to 3.93 eV.^33^ Abdelrazek *et al.*^34,35^ and Barzic *et al.* studied the blends of PVA/PVP (50/50) with different amounts of LiBr. They observed that *E*_g_ reduced from 5.10 to 4.40 eV.^35^ The *E*_g_ value of PVA can decrease from 3.448 eV to 2.605 eV in a system containing 2 wt% barium titanate. Moreover, the PVA doped with LiAsF_6_ showed a band gap reduction from 5.76 to 4.7 eV.^36^ An intensive literature survey revealed that the optical band gap of PVA did not change noticeably. In the current study, the metal complex was examined as an alternative to all the traditional approaches examined in the literature. The advantages of this approach are that it is environmentally friendly, economically feasible, and a moderate method for preparing the Zn-metal complexes, including an uncomplicated purification process, high product output, and quick reaction times.^37,38^ The literature review has shown that it is difficult to decrease the optical band of PVA to an appropriate value to be eligible for various applications. The present research investigates PVA's optical and structural characteristics incorporated with the Zn-metal complex. The current study's findings suggest that using environmentally friendly methods to create metal complexes is innovative and unique in reducing the optical band gap of biopolymers. This could have significant implications for photonics and optoelectronic devices and applications. This work employed numerous models and methodologies to accurately compute the optical band gap.

## 2. Methodology

### 2.1. Materials

Molecular weights of 12 000–18 000 were supplied by Sigma-Aldrich for the PVA powder. HOPKIN & WILLIAMS Zn(CH_3_COO)_2_ was also provided. The GT leaf was acquired from a marketplace located in London.

### 2.2. Metal complex preparation

**Scheme 1:**
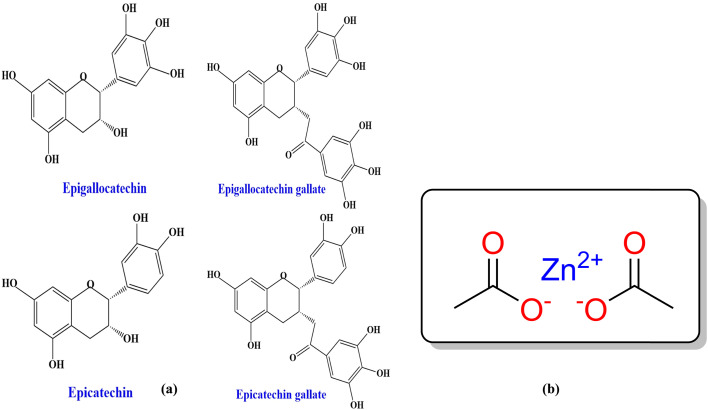
(a) Major green tea polyphenols and (b) structure of zinc acetate Zn(CH_3_COO)_2_.

A 22.8 g of Green Tea GT leaf and 800 mL of water (D.W.) mixture were prepared at approximately 95 °C without sunshine. After standing for thirty minutes, the extracted green tea solution was filtered (W. man paper 41, cat. no. 1441) with a pore radius of 20 μm comprehensive residue removal. Within an individual flask, 200 mL of D.W. was diluted by dissolving 10 g of Zinc Acetate Zn(CH_3_COO)_2_ salt. The structure of zinc acetate and the main catechins found in tea leaves are shown in Scheme 1a and b. It produced polyphenols (PPHs) in green tea (GT); the Zn^2+^–PPHs metal complex was then created by adding zinc acetate into the extracted green tea solution at 70 °C and stirring for sixty minutes. At the bottom of the tube, the extract solution's color changed from light green to dark green, confirming that Zn^2+^–metal ions and PPHs had complexed.

When the green tea solution progressed from light green to dark green and a cloud of precipitate developed at the bottom of the beaker, it was a sure sign that the Zn^2+^–polyphenol complex had formed. The complex mixture was allowed to settle down at room temperature. These complexes were extracted in 200 mL; after five days of washing, the Zn^2+^–PPHs metal complexes with D.W. Simplified PVA composite samples filled with Zn^2+^ metal complex were cast using the solution cast technique.

### 2.3. Sample preparation

The solution casting method produced composite samples using PVA doped with a Zn^2+^–polyphenol combination. The PVA solution, acting as the main polymer material, was created by thoroughly combining 5 g of pure PVA powder (with an average molecular weight of 18 000 g mol^−1^) with 250 mL of distilled water at 90 °C. The mixture was stirred for approximately one hour using a magnetic stirrer, resulting in an evenly distributed solution. It was then allowed to cool to room temperature. In 9 mL increments, different quantities of the zinc metal complex (Zn-metal complex) solution ranging from 0 to 36 mL were incorporated into the identical host polymer PVA solution. The solutions produced (extracted green tea and zinc acetate) were agitated for around 40 minutes. PVZMC0, PVZMC1, PVZMC2, PVZMC3, and PVZMC4 were utilized to represent 0 mL, 9 mL, 18 mL, 27 mL, and 36 mL of the Zn-metal complex, respectively, of the loaded metal complex solution.

**Scheme 2:**
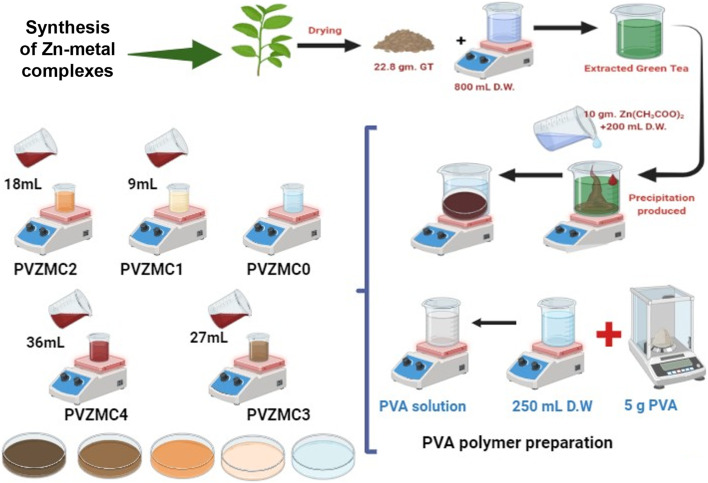
Abstract of the PVA and PVA/Zn-metal complex preparation methods.

The solution cast method created composite films from PVA impregnated with a foreign substance Zn^2+^–polyphenol complex. The mixture's contents were put into Petri dishes and left to dry at room temperature. Finally, the homogeneous solutions were cast into plastic Petri dish plates, and the water in this homogeneous solution gradually evaporated during the drying process at room temperature. The samples are ready for characterization after two weeks. Table 1 lists the pure polymer and doped PVA with a coordination metal complex. The methodology employed for preparing the metal complex (Scheme 2).

**Table 1:** Composition of PVA-green tea solution films

Identifying a sample	PVA (g mL^−1^)	Metal complex solution (mL)
PVZMC0	1/50	0
PVZMC1	1/50	9
PVZMC2	1/50	18
PVZMC3	1/50	27
PVZMC4	1/50	36

## 3. Characterization techniques

### 3.1. X-ray diffraction

The structural characterization and impact of the polymer complexation materials were evaluated using X-ray diffraction (XRD). The X-ray diffraction (XRD) patterns were obtained using a PANalytical X-ray diffractometer (model: 40 mA/40 kV) produced by PANalytical in Almelo, Netherlands. This study used a monochromatic X-ray beam with a wavelength of 1.5406 Å, specifically CuK_α_, to analyze the studied materials. The glancing angle (2*θ*) ranged from 5° to 50°, with a step size of 0.1°. All measurements were conducted at an ambient temperature.

### 3.2. FTIR study

The spectrophotometer operated in the range of 4000–400 cm^−1^, with a resolution of 4 cm^−1^ and 32 scans were accumulated. The samples were produced as KBr pellets, with a sample-to-KBr ratio of 1% w/w. A pure KBr disk was used as a reference.

### 3.3. UV-vis measurement

The UV-visible spectrometer is a scientific apparatus employed to quantify the absorption and transmission of light in liquid and solid samples within the ultraviolet (UV) and visible portions of the electromagnetic spectrum. The pure PVA and composite PVA were analyzed using UV-vis spectroscopy for absorption with a Jasco V-570 UV-vis-NIR spectrophotometer (manufactured by Jasco, Japan, model SLM-468). The spectra were recorded in absorbance mode within the 190–1100 nm wavelength range. The thicknesses of the samples were measured in the range of 100–145 μm.

## 4. Results and discussions

### 4.1. Structural analysis

**Fig. 1:**
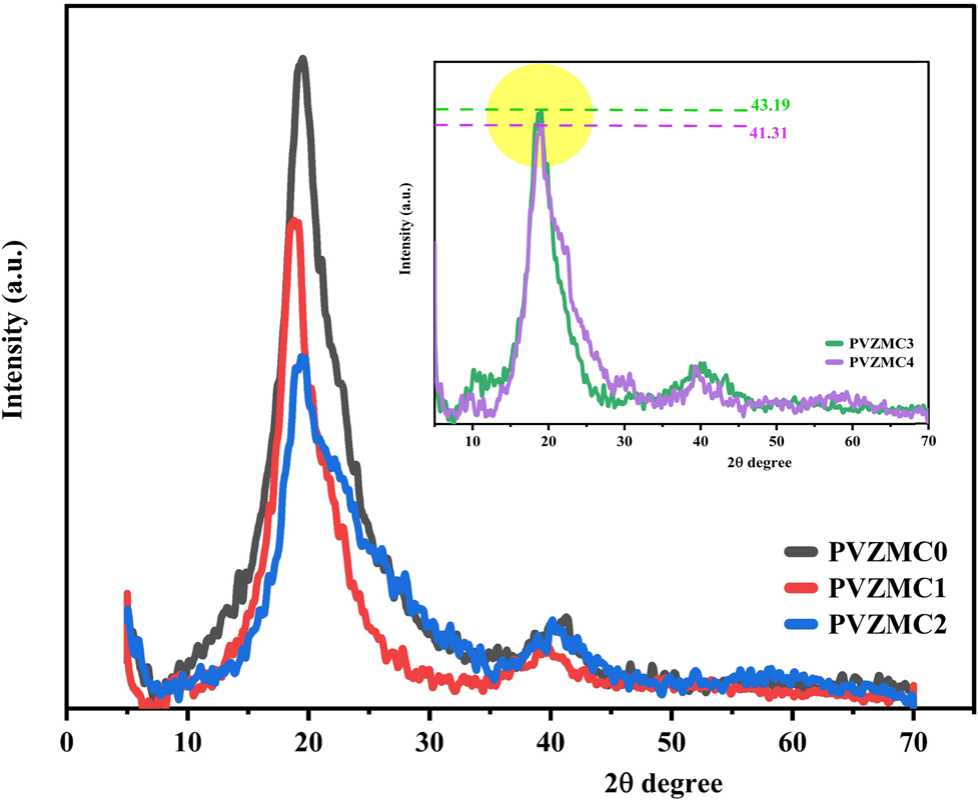
XRD pattern of films made of pure PVA and between PVA and Zn-metal complexes.

Fig. 1 displays the patterns created by XRD of the Zn^2+^–PPL complex, pure PVA, and the PVA/Zn-metal complex. According to Achilles *et al.* (2018), larger peaks are associated with the amorphous phase, whereas sharper peaks distinguish the crystalline phase.^39^ A sharp peak with a wide distribution centered at 19.54 was detected in the X-ray diffraction (XRD) of pure PVA (see Fig. 1). However, in the PVZMC1, PVZMC2, PVZMC3, and PVZMC4 composite films, the peak corresponding to pure PVA (19.54) exhibited a broader shape and decreased intensity. The expanding shape of the peak demonstrates the lack of a defined structure in the complex system. The interpretation of these data might be based on Hema M. *et al.*^40^ The guideline establishes a direct relationship between the height of the highest point and the degree of crystallinity. The inset plots in Fig. 1 represent the XRD curves of PVZMC3 and PVZMC4, which have similar intensity values. The purpose of the inset is to distinguish between the two samples.

The XRD spectra of the polymer composite broadening films show a notable decrease in intensity and signal inside the range of 2*θ* = 40–55, which is considered the most valuable area. This phenomenon suggests a rise in the amorphous configuration inside the resulting hybrid films. The observed change is most likely due to substituting the typical intermolecular hydrogen bonding seen in pure polymers with unpredictable intermolecular hydrogen bonding in composite films. Hydrogen bonds, which are directed intermolecular contacts, substantially impact the conformation of polymers and their optical and physical properties. As the concentration of the Zn-metal complex increases, the crystallinity of the PVA decreases. The XRD measurements verified the presence of distinct complex coordination between PVA and Zn-metal complexes. The strong interactions between the polar functional group, OH of PVA, and metal complex OH and NH groups reveal the excellent compatibility and miscibility of the polymer and metal complex components. This complex formation reduces the degree of crystallinity of the composite.^41^

### 4.2. Fourier transform infrared (FTIR) analysis

**Fig. 2:**
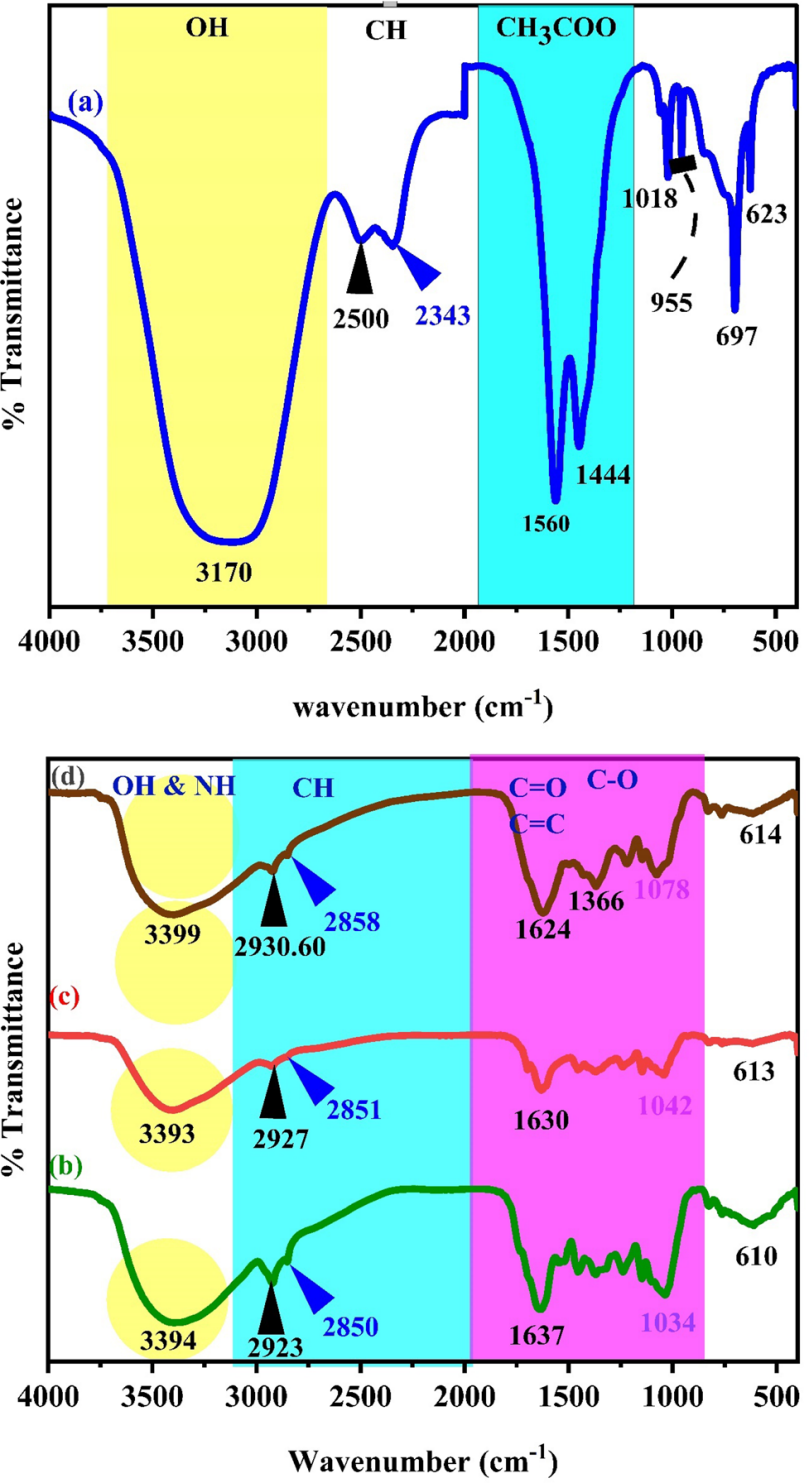
FT-IR spectra of (a) zinc acetate, (b) green tea powder, (c) extracted green tea dye, and (d) Zn-metal complex.

Fig. 2a–d illustrates the FTIR bands of the zinc acetate salt, green tea powder, extracted green tea dye, and Zn-metal complexes, respectively. From Fig. 2a, the final two bands are detected in the range of 1600–1350 cm^−1^, which acts as a distinctive characteristic of the acetate anion group present in the compounds. The carboxyl acetate groups in basic metal salts typically form coordination bonds with specific cations.^42^ The vibrational modes of the carbonyl group (C=O) are influenced by the coordination formed by the inserted acetate. Acetate with zinc is associated with three well-known coordination types. The two bands detected at 1560 and 1444 cm^−1^ were observed phenomena that can be attributed to the asymmetric stretching vibrations of the C=O bond.^43^

Fig. 2b and c display the transmission spectrum as a function of wavenumber for the green tea powder and extracted GT dyes, specifically in the 400 to 4000 cm^−1^ range. The broad peak presence of phenolic hydroxyl groups is responsible for this peak. Each infusion displayed a distinct and wide peak between 3000 and 3600 cm^−1^, suggesting the presence of hydroxyl groups (alcoholic substances or phenols), C–H stretching (alkenes or arenes), and N–H stretching (amine groups) that intersected. The infrared spectrum of the GT sample showed absorption peaks in specific regions: between 2850 and 3600 cm^−1^ (which indicates the stretching of O–H bonds in carboxylic acids and C–H bonds in alkanes), 1500–1750 cm^−1^ (indicating the stretching of C=O bonds in carbonyl acids and esters, bending of N–H bonds in amides, stretching of C=C bonds in alkenes and aromatic groups, and stretching of C=N bonds in aromatic groups), 1250–1500 cm^−1^ (indicating the stretching of C–O bonds in carboxylic acids, bending of C–H bonds in alkanes, and bending of O–H bonds in alcohols/phenols), 930–1200 cm^−1^ (which indicates the stretching of C–O bonds in alcohols/phenols/esters, stretching of C–N bonds in amines, and bending of =C–H bonds in alkenes), and numerous points in the region 800–930 cm^−1^ (indicating the bending of =C–H bonds in alkenes and bending of C–H bonds in aromatic groups) in the GT sample. The GT had a prominent and wide peak across the 3000–3600 cm^−1^ range.^44–47^ The FTIR spectra of powder GT and GT dye extracts are almost the same. Widatalla *et al.*^48^ also discovered that GT dye has distinct peaks at specific wavenumbers: 3566.3, 2918, 2850.7, 1653.9, 1384, 1107, 1053, and 997.2 cm^−1^. These peaks correspond to O–H bond stretching in alcohol present in polyphenols and amines, as shown in Table 2.

**Table 2:** Table and graphic representation of the FTIR peak of zinc acetate, GT powder, extracted GT, pure PVA, and composite PVA/Zn-metal complex films

Samples	OH & NH	CH	C=O	C–O	C–O–C	(CH_3_COO)_2_
Zn(CH_3_COO)_2_	3170	2500				1560
		2343				1444
G.T. powder	3394	2923	1637	1034		
		2850				
Extracted G.T.			1630	1042		
Zn-metal complex			1624	1078		

**Composite samples**						
PVZMC0	3302	2941	1729.86	1086.50	1246	
		2910.36				
PVZMC1	3299	2943.40	1722.30	1086	1244	
		2913.23				
PVZMC2	3295	2941.96	1721.36	1086	1242.50	
		2911.97				
PVZMC3	3295	2940.56	1718.60	1084	1239.70	
		2910.36				
PVZMC4	3289	2940.53	1713.75	1080	1239.70	
		2910.36				

The stretching of carbon–hydrogen (C–H) bonds in alkaline substances and carboxylic acids, the presence of carbon–carbon double (C=C) bonds in aromatic compounds and primary amide groups in proteins (–CO–NH_2_), the stretching of carbon–oxygen–carbon (C–O–C) bonds, and the bending of C=C bonds were observed. The aqueous extract of GT (green tea) was analyzed for phytochemicals, revealing the presence of various polyphenols, such as gallic acid (GA), gallocatechin (GC), catechin (CE), and epigallocatechin (ECC), together with proteins, flavonoids, saponins, and glycosides. Catechins, a type of polyphenol, make up the majority of GT, accounting for 24–36% of its dry weight. However, based on Fig. 2d of the Zn-metal complex, the absence of acetate peaks in the range of 1350–1600 cm^−1^ indicates that no acetate is present in the metal complex. The peaks observed at 1366 cm^−1^ and 1624 cm^−1^ in Fig. 2d are not due to acetate salt because these peaks exist in the GT dye, and the development of metal complexes is now established.

**Scheme 3:**
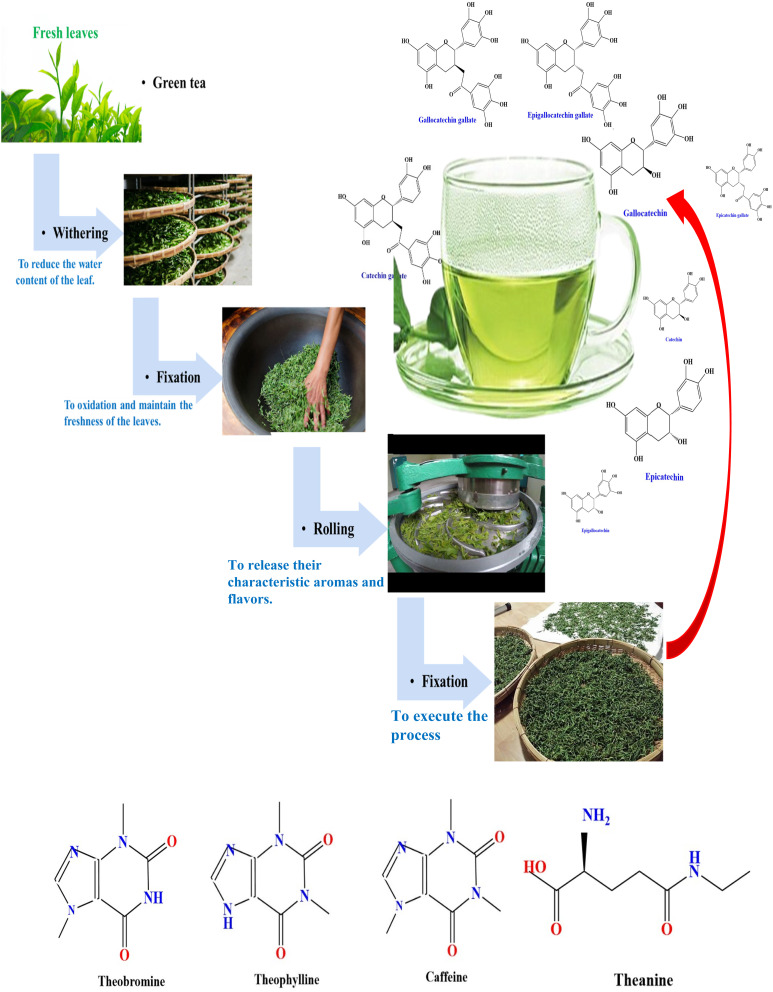
Components present in extracting the green tea dye enriched with NH and C=O functional groups.

**Scheme 4:**
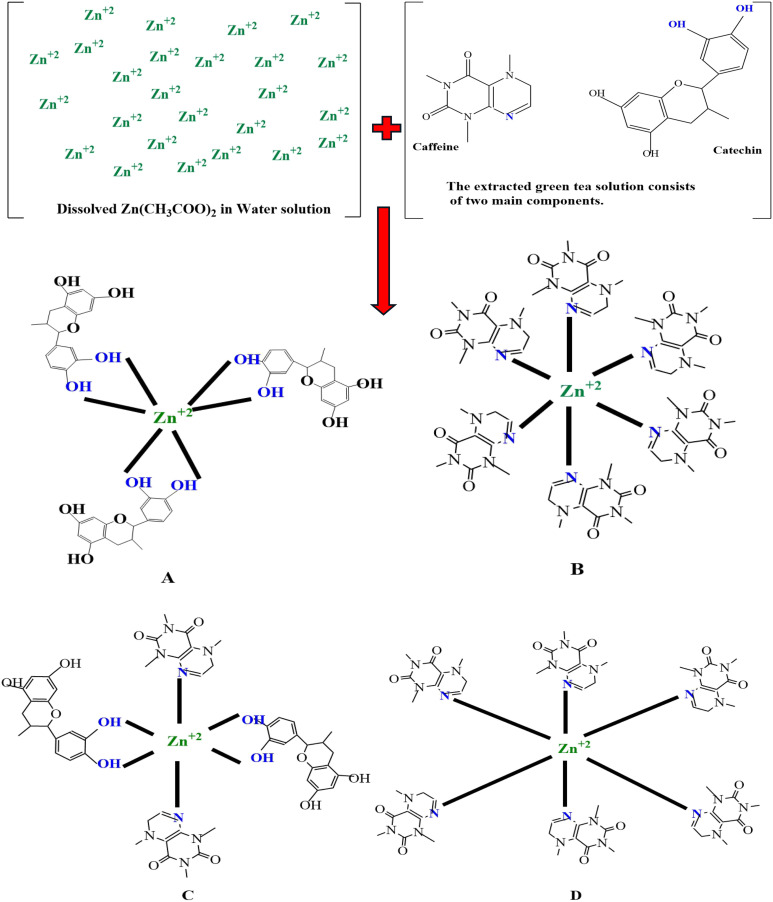
The proposed structure for producing metal complexes involves binding ligands Caffeine and catechin to central metal ions, Zn^2+^. The compounds mentioned are (A) a bidentate complex of [Zn(C_15_H_14_O_6_)_3_], (B) a monodentate complex of [Zn(C_8_H_10_N_4_O_2_)_6_], (C) a complex with both bidentate and monodentate [Zn(C_15_H_14_O_6_)_2_(C_8_H_10_N_4_O_2_)_2_], and (D) monodentate [Zn(C_8_H_10_N_4_O_2_)_6_] structure.

Fig. 2d illustrates the characteristic frequencies of Zn-metal complexes in both the infrared (IR) and far infrared (far IR) regions. The infrared spectrum of the bound ligand has a distinct peak at 2930.60 and 2858 cm^−1^, which is attributed to the presence of methyl (CH). A wide band is also observed at 3411 cm^−1^, corresponding to the hydroxyl group (OH) in its phenolic form. Nevertheless, the infrared spectra of the Zn-metal complex indicate the existence of a novel wide band in the range of 3000–3750 cm^−1^, which can be ascribed to a combination of (NH) and (OH) vibrations originating from the coordinated or lattice water. The alterations in stretching frequencies associated with (NH) and (OH) cannot be discerned due to the merging of that region with m(OH) of water, resulting in a broad band in the complexes.^49^ A typical instance of a C=O stretching vibration, commonly observed in ketones and aldehydes, occurs at around 1624 cm^−1^. The compound's molecular structure is characterized by very particular and unique patterns in the ∼1500–400 cm^−1^ range. Nevertheless, the positions of peaks might differ because of various factors, including the molecular surroundings, the way the sample is prepared, and the instrument's parameters. This emphasizes the significance of referring to dependable databases and literature to ensure precise peak assignments.^50^ In the present situation, specific peaks are observed within the range of 600–850 cm^−1^ during the creation of the Zn-metal complex. However, in the case of green tea, there is no discernible peak in the range mentioned above. Following the complexation process, a band emerged, accompanied by the discovery of additional bands at 830, 765, and 614 cm^−1^ in the zinc metal complex. Fig. 2a shows a distinct peak corresponding to the Zn(ii) at 697 cm^−1^, which displays higher intensity. However, in Fig. 2d, the strength of this peak vanished due to the formation of coordination between zinc and polyphenols in GT, facilitated by the acceptance of a pair of electrons by the zinc metal ion. This is related to the metal ion being more stable. The prepared dye and proposed Zn-metal complex structure are illustrated in Schemes 3 and 4, respectively. The metal complex leads to a decrease in the frequency and intensity of most peaks in the FTIR pattern of the Zn-metal complex compared to the corresponding GT ligands. This decrease depends on factors such as the specific metal cation mass, the kind of ligands (GT), and the oxidation state of the metal. In PVA polymers, intramolecular hydrogen bonding occurs between OH groups on the same polymer chain, while intermolecular hydrogen bonding occurs between the OH functional groups in different polymer chains. Introducing the Zn-metal complex into the PVA polymer disturbs the intermolecular hydrogen bonding among polymer chains, thereby reducing the extent of intermolecular hydrogen bonding. Incorporating the Zn-metal complex into PVA polymer creates a powerful link between the metal complex's OH or NH functional groups and the hydroxyl OH group of PVA. This interaction creates a space between these functional groups and reduces the degree of crystallinity.^51,52^

**Fig. 3:**
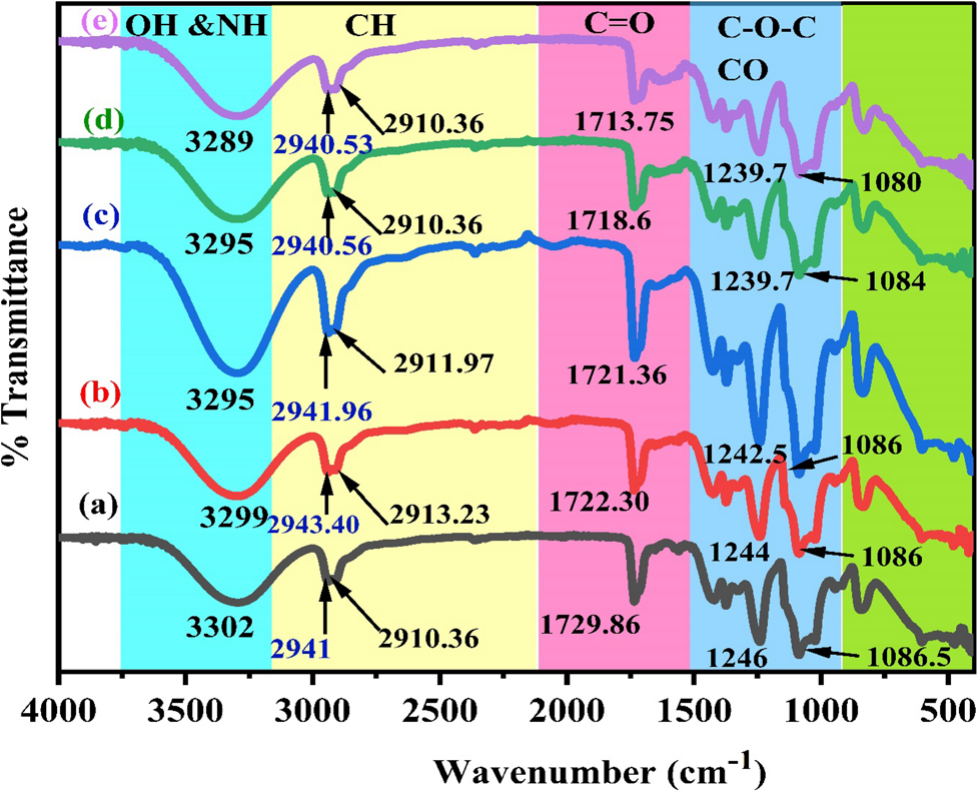
FTIR spectra of polar polymer PVA with zinc metal complexes in the region 400–4000 cm^−1^ for pure (a) PVZMC0 (b) PVZMC1 (c) PVZMC2 (d) PVZMC3 and (e) PVZMC4 films.

Fig. 3a depicts the FTIR spectrum of pure polymer PVA. Upon comparing the spectral properties of complex PVA with those of pure PVA, GT, and Zn-metal complex, the following modifications were observed. The frequency with which the OH groups in PVA form intermolecular hydrogen bonds, initially observed at 3302 cm^−1^, is moved to 3289 cm^−1^ when PVA forms a complex film. Furthermore, the CH stretching of CH_2_ in pure PVA, which is initially seen at 2941 cm^−1^, is shifted to 2943.40, 2941.96, 2940.56, and 2940.53 cm^−1^.^53^ The peak observed at 1729.86 cm^−1^ refers to the stretching vibration of the C=O bond in the carboxylic groups (COOH) in pure PVA. The stretching modes of C–O and C–C are responsible for the peaks at 1087 and 1571 cm^−1^, respectively, in the PVA backbone. The stretch C–C vibrations of the planar zigzag structure of the carbon backbone show moderate absorption at 836 cm^−1^. The peak at 651 cm^−1^ corresponds to the wagging mode of (OH) groups.^54^

The FITR spectrum of the polar polymeric metal complex reveals the presence of additional bands. Nagesh *et al.* observed that in the zinc complexes, the presence of Zn–nitrogen and Zn–oxygen bonds may be identified by low-intensity bands ranging from 420 to 450 cm^−1^.^55^ Although there are many possibilities, a general structure for the complexes is proposed based on the literature. As a result of extending the C=O bond in the acetate group of PVA, a peak at 1729.86 cm^−1^ was noted. This peak is displaced to 1722.30, 1721.36, 1718.60 and 1713.75 cm^−1^ in the polymer complexes, respectively, as depicted in Fig. 3b–e curves of the polymer composites, respectively, as shown in Table 2. In the pure PVA, the peak at 1087 cm^−1^ exhibited a decreased amplitude and shifted to 1086, 1086, 1084, and 1080 cm^−1^ when doped with 9, 18, 27, and 36 mL of Zn-metal complex, respectively, in the polymer complex. The polymer with a metal complex exhibits peaks in the 400 to 700 cm^−1^ range, which is attributed to the presence of Zn cations. This observation provides evidence of interactions between the polymer and the metal complex.^56^

Fig. 3 depicts the FTIR spectra of pure PVA and PVA : Zn-metal complex composites. The hydroxyl groups exhibit broad and intense OH stretching absorption. The absorption peak of pure PVA is observed at 3302 cm^−1^. Curiously, the intensity of this peak varies and decreases as the Zn-metal complex increases. The intensity of this peak decreased significantly when 36 mL of Zn-metal complex was present, causing a shift to 3289 cm^−1^. The CH asymmetric stretching vibration band in pure PVA occurs at 2941 cm^−1^, as illustrated in Table 2. However, in the doped samples, there is an apparent change to 2940 cm^−1^, followed by a considerable decrease in the intensity of this band.

### 4.3. Optical characterization

#### 4.3.1. Light-matter interaction and absorption coefficients

Ultraviolet-visible (UV-vis) spectroscopy is a valuable tool in our study, allowing us to investigate the optical characteristics and identify electronic transitions in polymer–metal complex films.^7^ The absorption spectrum of green tea dye shows a significant absorption peak at 671 nm. In contrast, the absorption spectrum of the Zn-metal complex displays the same peak, albeit with reduced intensity. This observation suggests a positive interaction between Zn acetate and green tea.^57^ Green tea, a rich source of catechins and caffeine, contains these compounds as the main functional groups of polyphenols, accounting for approximately 20–30% of the powder of green tea. In addition, it includes many more polyphenols, such as gallic acid, the main polyphenolic acid present in tea, and minor amounts of alkaloids, such as caffeine. The spectra displayed absorption peaks at 407 nm and 671 nm, within the visible range of 400–800 nm. The tea leaves mostly absorb light within the visible spectrum, particularly in the violet range (380–435 nm) and the red range (622–770 nm), giving them a green color.^58,59^ Mahasen *et al.*^60^ showed green tea peaks at 672 nm. The absorption spectra peaks observed at 324, 363, and 381 nm in the Zn-metal complex can be attributed to the π → π* and n → π* transitions. The absence of the band d–d transition in the Zn-metal complex can be attributed to electron pairing in the main orbital. Conversely, the combination accelerates the transfer of charges between the ligand and the metal in both directions. Furthermore, it is crucial to consider the possible electronic transition and orbital contributions of both ligands and their metal complexes.^61^

**Fig. 4:**
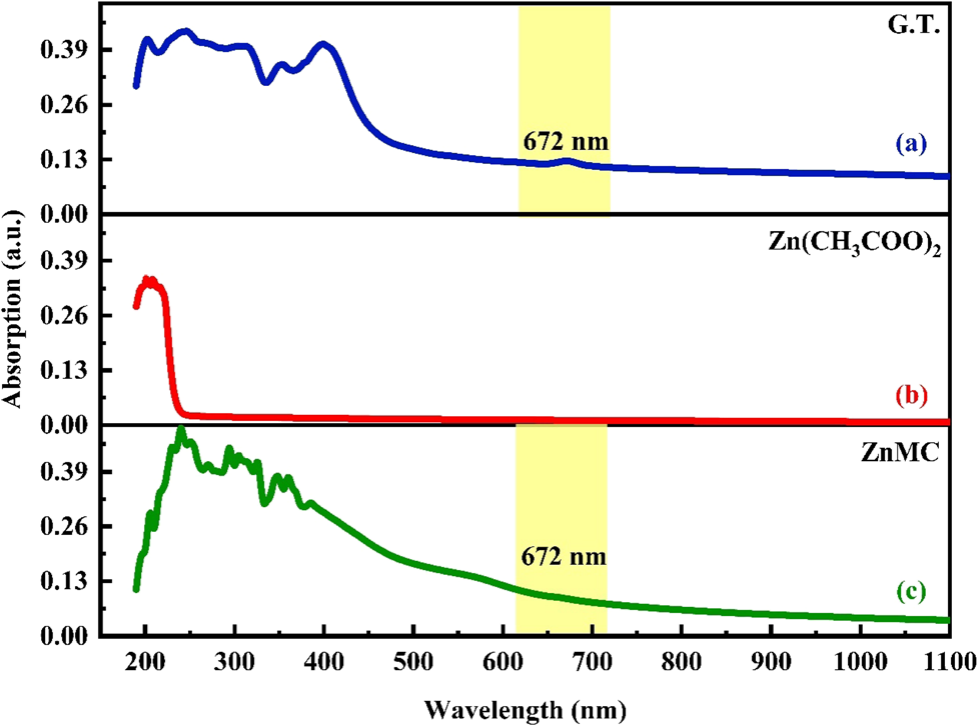
Absorption spectra for (a) pure G.T., (b) zinc acetate salt, and (c) Zn-metal complex samples.

As shown in Fig. 4, decreasing the concentration (diluted) of a solute in a solution is referred to as dilution, and it is almost often accomplished by simply mixing with an additional solvent (in this process, D.W.). Fig. 4b illustrates the UV-vis. of zinc acetate ranging from 325 nm to 700 nm. Interestingly, no notable peak was observed in the absorbance of zinc acetate inside the visible area. No substantial fluctuations in the absorption properties of zinc acetate were detected in this area. However, based on Fig. 4c, the findings demonstrated that combining zinc acetate and green tea G.T. produced a Zn-metal complex.^62,63^

The two (Fig. 4a and c) exhibit exponential curves in the visible region. UV-visible absorption spectroscopy can be utilized to investigate the interaction between metal complexes and PVA polymers. The investigation involved adding different concentrations of Zn-metal complex to PVA to observe how the position of the absorption bands changed due to the interaction between the metal complex and PVA. In addition to varying concentrations of Zn-metal complex to PVA, a noticeable shift to a high wavelength occurred in the absorption position; due to the color of the metal complex, this means that there is a good interaction between the metal complex and PVA.

During the process of absorption, an electron is elevated from a lower energy band to a higher energy band by the interaction with a photon possessing a specific energy concept of absorption, which is quantified by a coefficient (*α*), the ratio of the initial light intensity to the light intensity after a reduction.^64^ Using Lambert Beer's relation, the absorption coefficient (*α*) for a specific film thickness (*d*) was computed. An essential variable in this context is the optical absorbance (*A*). The correlation between the energy of the incident photons (*hv*) and the coefficient of absorption (*α*) was shown by a drawn graph. The linear section of the *α-hv* curves was extrapolated to the horizontal axis to calculate the absorption edges of the created samples.^65,66^

**Fig. 5:**
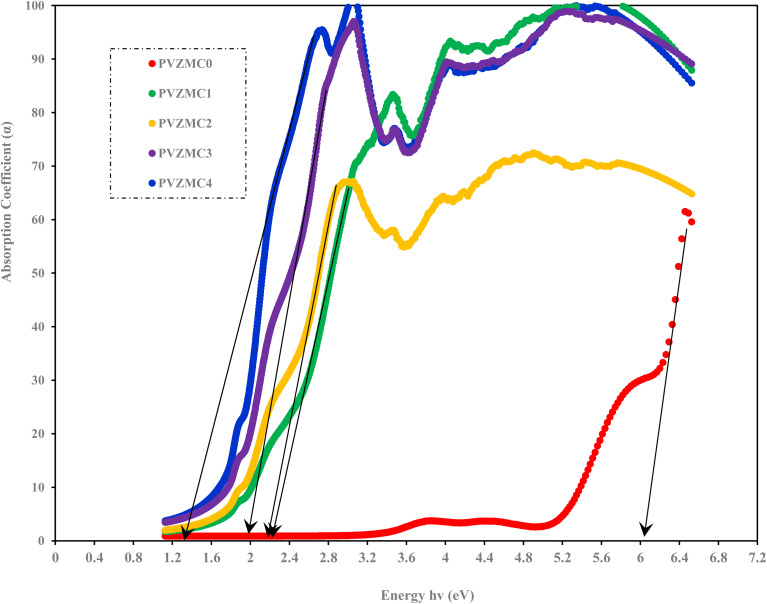
Absorption coefficient *vs.* incident energy for pure PVA and PVA/Zn-metal complex films.

By incorporating Zn-metal complex molecules into the polar PVA polymer, the bandgap of the PVA-doped material was significantly reduced, resulting in a downward change in the location of the valence band. Furthermore, the electronic states at the Fermi level of the Zn-metal complex and PVA exhibit hybridization, indicating that combining Zn-metal complex molecules substantially impacts the electronic states below the Fermi level. Kalarani *et al.*^67^ utilized the Zn-metal complex of a bidentate Schiff base ligand (L) 2-((1H-benzo [*d*]imidazole-4ylimmino)). It was found that the HOMO and LUMO orbitals of the L-Zn^2+^ combination were mainly located on both the ligand and metal systems, and the band gap was 1.76 eV. This finding closely aligns with the energy gap of our hypothesized Zn-metal complex, as depicted in Fig. 5.

As illustrated in Fig. 5, the observed phenomenon is that the absorption edge of the hybrid films is moved towards lower photon energy compared to pure PVA, providing empirical support for the indispensability of metal complexes in doping functional polymers. The absorption edge values for metal complex PVA films with contents of 0, 9, 18, 27, and 36 mL were determined to be 6.05, 2.32, 2.27, 1.98, and 1 eV, respectively. The formation of novel localized states within the gap could cause these occurrences. This observation suggests that the integration of the Zn-metal complex into the PVA matrix results in a decrease in the band gap energy.^68–70^1
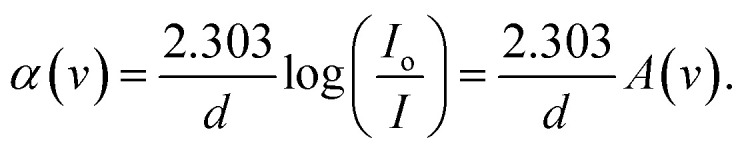


In the previous context, the amount of light that hits the object is depicted by the symbol *I*_o_; *I*_T_ represents the transmitted light intensity, and the symbol (*d*) defines the thickness of the sample. This study investigates the relationship between the variation in *α*(*ν*) and photon energy (*hν*) in pure polymer PVA and PVA films doped with Zn-metal complexes samples.^64,71^

**Fig. 6:**
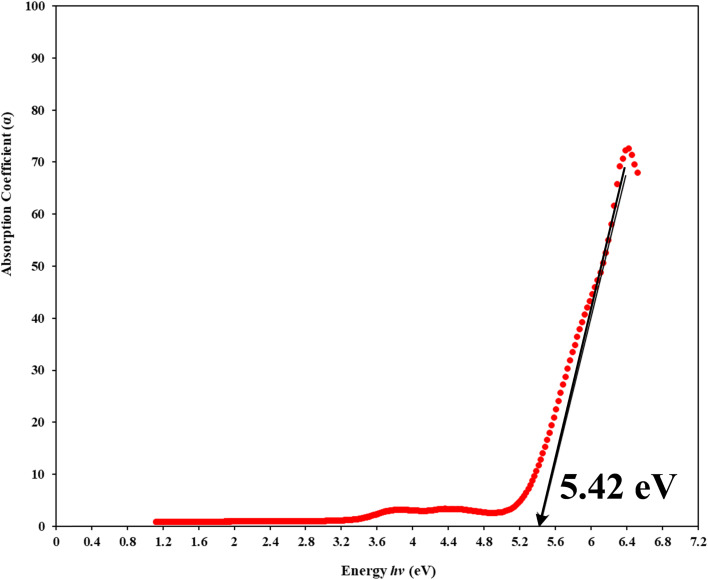
Absorption coefficient *vs.* incident energy for PVA/(CH_3_COO)_2_ polymer electrolyte (PE) film. It is clear that the optical properties of the polymer: the salt system is not interesting because salts cannot provide enormous electrons into the polymer matrix.

To understand how the variations between Zn-metal complexes and Zn polymer electrolytes affect the energy band gap, a solution casting process was used to make a polymer film of PVA/ZnAc electrolytes. This phenomenon is shown in Fig. 6. As depicted in Fig. 5 and 6, metal complexes have a more significant impact on energy band gaps than polymer electrolytes because the edge of absorption of PVA films shifts from 6.05 eV to 5.42 eV. Aziz *et al.*^38^ presented novel strategies that combine a green method of manufacturing metal complexes with a host PVA polymer.

#### 4.3.2. Dielectric constants and energy band gap study

The values of the real and imaginary parts (*ε*_r_), and (*ε*_i_), respectively, of the dielectric constant were ascertained by employing the given formula and can be expressed as follows:

2 *ε*_r_ = *n*^2^ − *k*^2^.

The refractive index, denoted as (*n*), is associated with the actual velocity, and an extinction coefficient (*k*) is related to loss. The Fresnel formulas are utilized to determine the refractive indices of pure polymer PVA and composite PVA films. This is achieved by analyzing the reflectance, *R*, and the optical loss coefficient, *k*, which can be calculated using the formula *k* = *αλ*/4π*d*. Here, α represents the absorption coefficient, *λ* denotes the wavelength, and *d* denotes the thickness of the sample.3
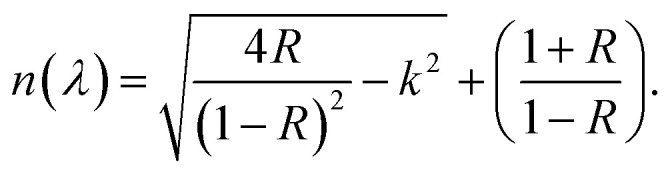


In the present study, practically, we can investigate the variations in the real (*ε*_r_) and imaginary (*ε*_i_) components of the dielectric constant for various films composed of (PVA) and PVA with a metal complex. Based on the analyses of (*n*) and (*k*), it is found that the magnitudes of the real components are greater than those of the imaginary parts. The refractive index of pure PVA increased from (1.1553 to 1.3569) by doped 36 mL Zn-metal complex. The optical data reveal an identical trend in the refractive index (*n*) and the real parts of the samples (*ε*_r_). The real portion (*ε*_r_) serves as an indicator of the degree of polarization. According to the reference, there is a positive correlation between the degree of polarization and the value of (*ε*_r_).^29^

It is possible to determine the actual component of the real part of the dielectric constant from a theoretical perspective:4
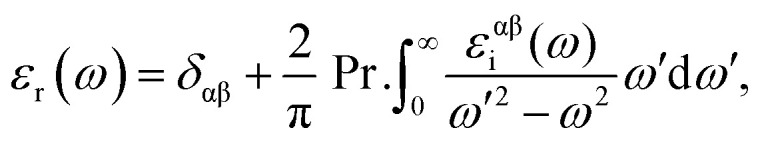
where Pr. is the main value of Cauchy. Practically, the determination of the real part of the dielectric constant (*ε*_r_) is simpler than in a theoretical situation. The results obtained from the experiment demonstrate that there is a positive correlation between the filler content and the increase in (*ε*_r_) values of PVA/Zn-metal complex films. The observed increase in the relative permittivity (*ε*_r_) of the metal complex samples can be ascribed to the higher density of the films resulting from an increase in filler content. This increase in density is caused by a continuous increase in the number of atomic refractions, which is related to an increase in linear polarization. The introduction of metal complexes in polymer composites can result in an increased packing density, potentially leading to the development of strong intermolecular bonding between the inserted functional group of the Z-metal complex and the –OH active functional groups of PVA through electrostatic interactions.^72,73^

**Fig. 7:**
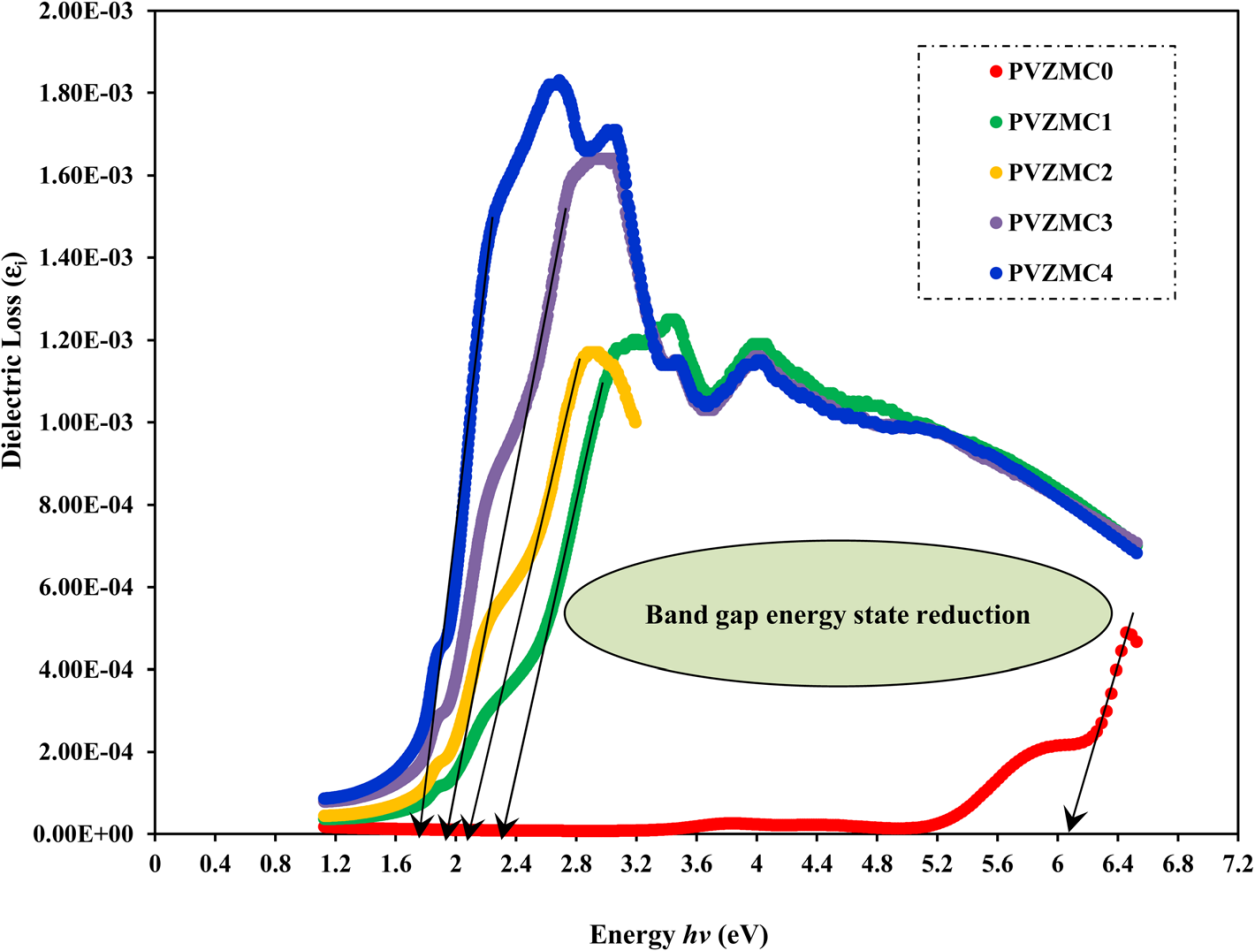
Optical *ε*_i_ spectra of pure PVA and PVA/Zn-metal complex films.

Fig. 7 illustrates the imaginary component of the dielectric *vs.* photon energy (*hv*). A previous study demonstrated a significant association between the band structure and optical characteristics of materials. The detailed dielectric function affects the optical characteristics of absorbing isotropic substances depending upon the wavelength. The imaginary dielectric function (*ε*_i_) is used to describe the electronic absorption of materials resulting from the interaction with an electromagnetic field, which induces dipole motion. The complex dielectric function is commonly utilized to provide insights into the optical characteristics.^9,74^ The complex dielectric function is a valuable parameter for precisely characterizing many optical features, such as the reflectivity ratio and absorption spectrum. Theoretically, the imaginary component of the dielectric function is determined by applying eqn (5), which considers interband transitions of active electrons between occupied (*i*_k_) and unoccupied electron states (*f*_k_).^21,73^5
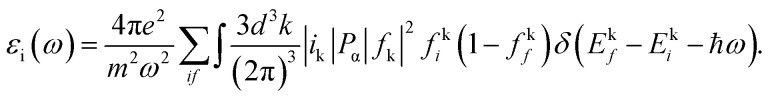


In eqn (5), the symbol *ω* represents the frequency of incident photons and *Ω* represents the crystal volume. The fundamental unit of electric charge is denoted as *e*, and the permittivity in the vacuum of space, denoted as *ε*_o_, is the respective term used in scientific discourse. The terms (*f*^k^_*i*_) and (*f*^k^_*f*_) denote the initial and final states of transition electrons, respectively. The electronic transitions between the occupied states *i*_k,_ and unoccupied states *f*_k_ wave functions cause photon absorption or emission. These transitions are accurately represented by the optical dielectric loss (*ε*_i_). The optical band gap computation is based on the extrapolated linear intersection of (*ε*_i_) in its place of (*hv*), as demonstrated. The following expression illustrates the practical relationship to determine the imaginary part of the optical dielectric constant. From the gained *n* and *k* data, the *ε*_i_ can be computed using the following equation:^9^

6 *ε*_*i*_ = 2*nk*.

Analyzing optical absorption spectra is crucial for determining the optical energy gap (*E*_g_) in organic and inorganic materials. In numerous amorphous materials, photon absorption follows the Tauc relation, which can be expressed as follows:^75^

**Fig. 8:**
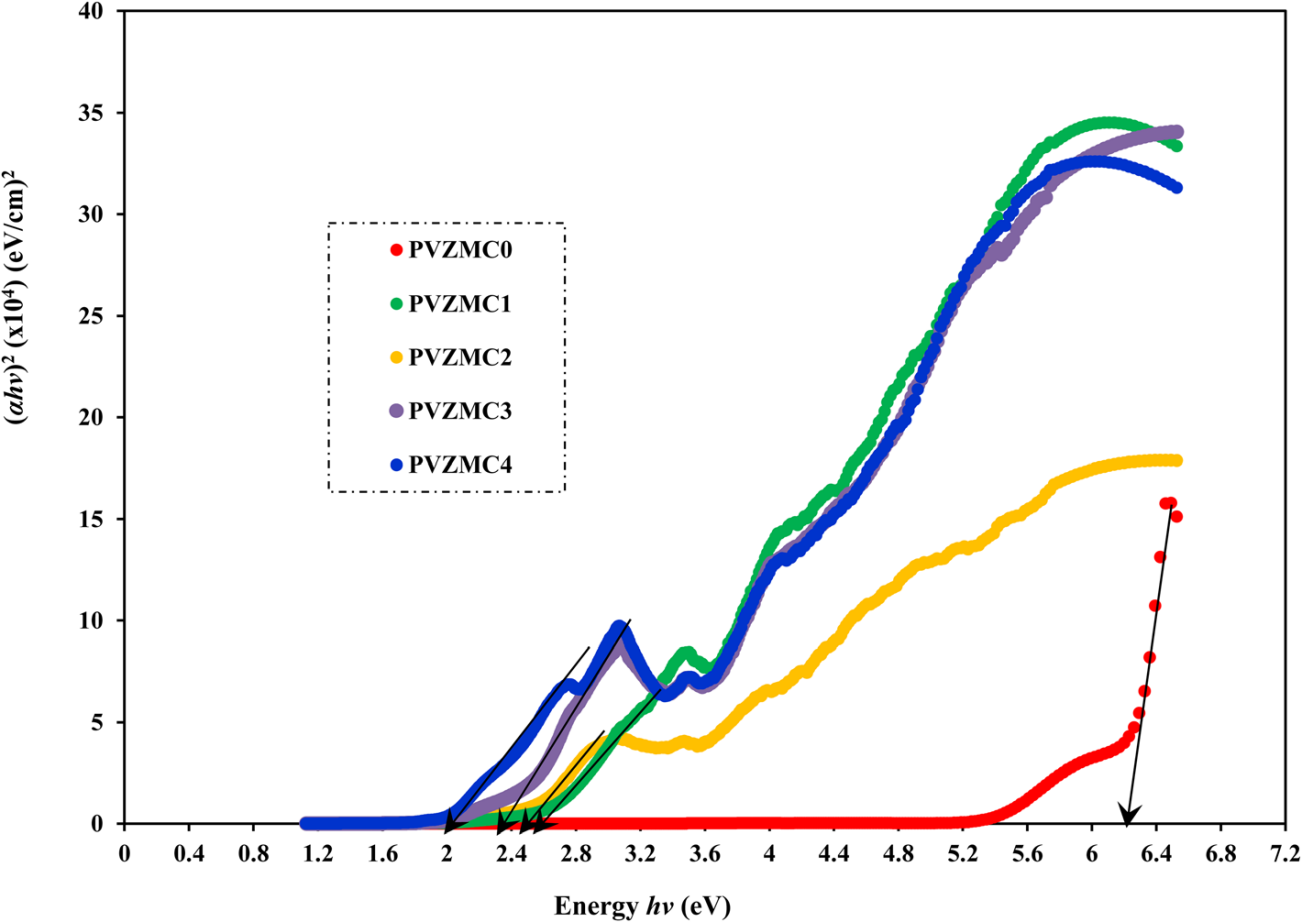
Direct allowed band gap energy plot of pure polymer PVA and composite PVA/Zn-metal complex films.

**Fig. 9:**
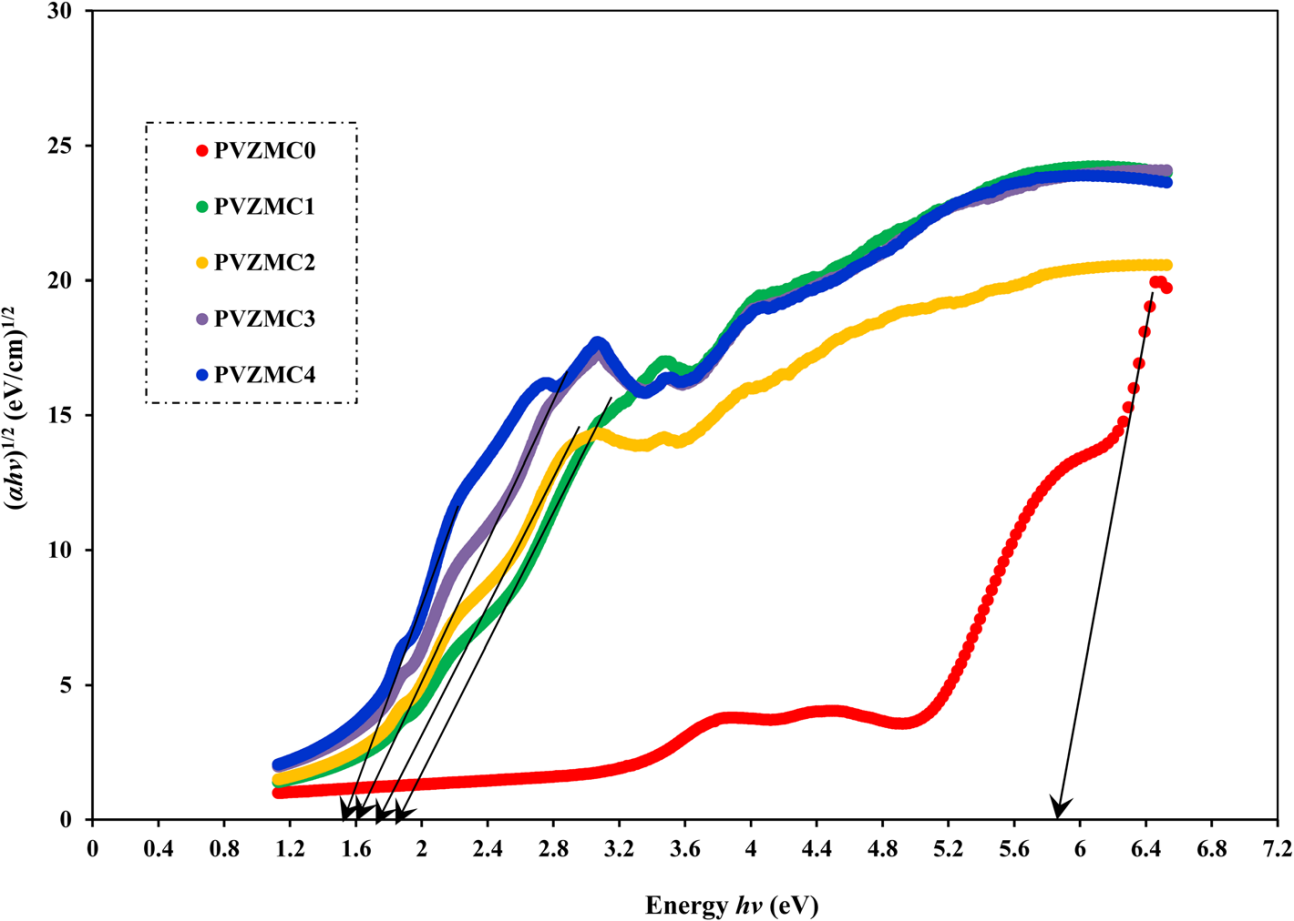
Indirect allowed band gap energy plot of pure polymer PVA and composite PVA/Zn-metal complex films.

**Fig. 10:**
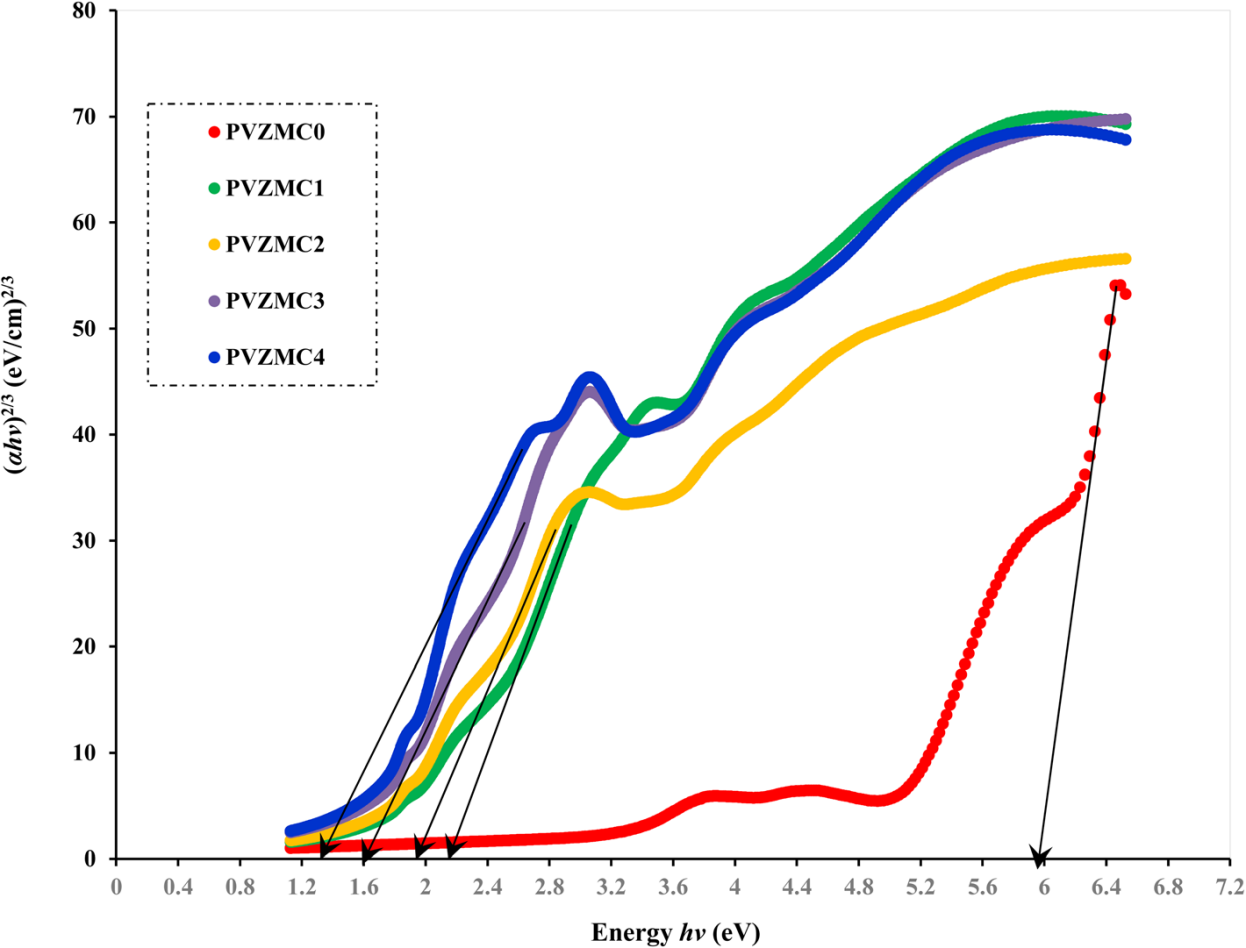
Direct forbidden band gap energy plot of pure polymer PVA and composite PVA/Zn-metal complex films.

**Fig. 11:**
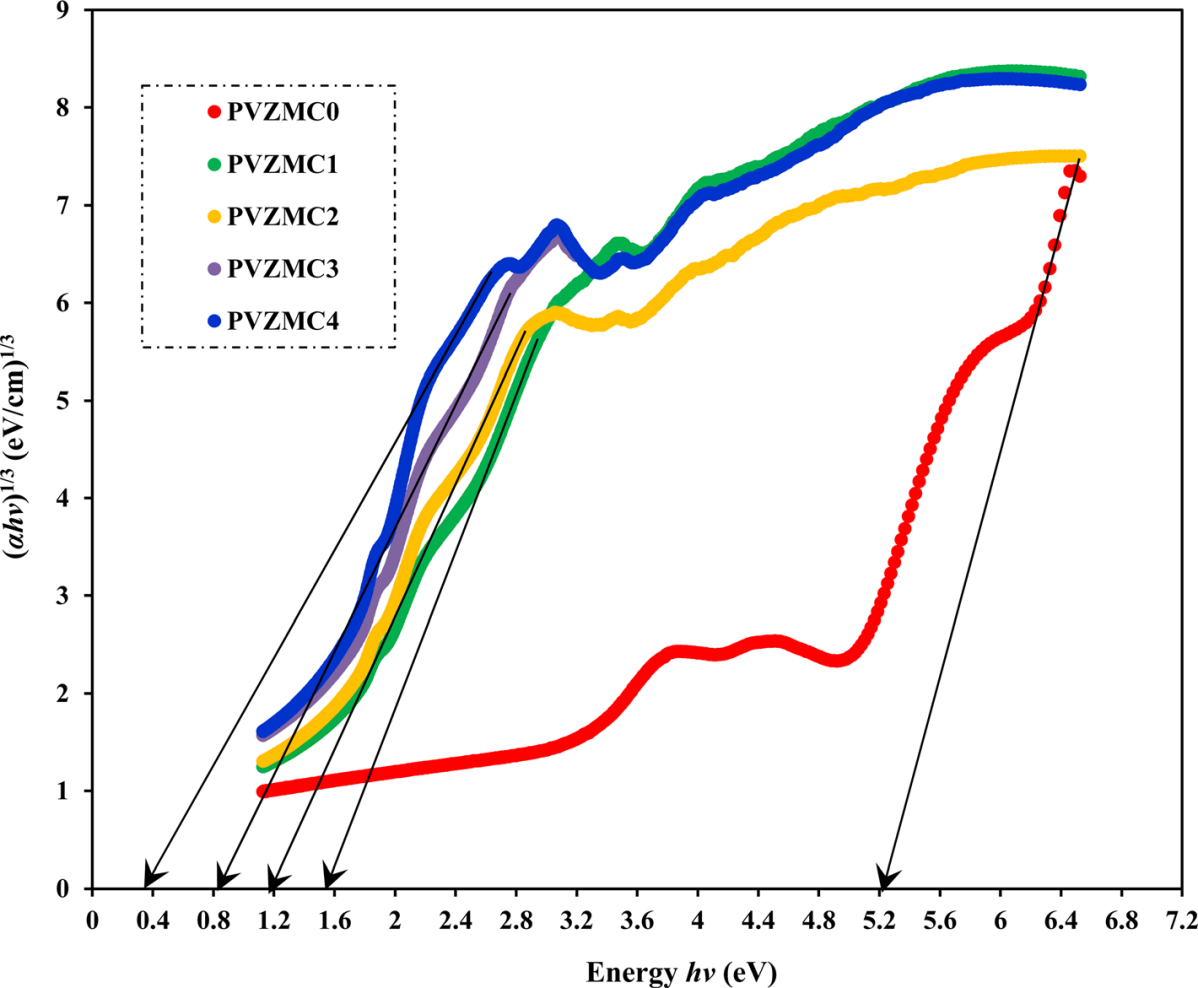
Indirect forbidden band gap energy plot of pure polymer PVA and composite PVA/Zn-metal complex films.

7 (*αhν*) = *B*(*hν* − *E*_g_)^*γ*^,

where *B* denotes the parameter that depends on the probability of inter-band transitions and *hv* is the energy of the incident photon. Moreover, the physical properties of the electronic transitions responsible for optical absorption can be determined by index *γ*. When electron transitions are directly allowed and indirectly allowed, the values of *γ* are 1/2 and 2, respectively. When *γ* is 3/2 or 3, it corresponds to direct and indirect forbidden transitions, respectively. To find the *E*_g_ value, we captured the extrapolated linear segment of the plot of (*αhν*)^1/*γ*^ against *hv* with the abscissa. Fig. 8–11 illustrates all possible values of *γ*, describing the type of transition associated with electrons that may cross the optical band gap. Based on previous studies,^76–78^ many localized charge carrier levels or trapping sites are inside the constrained band gap. Thus, a reduction in the optical energy band gap is expected. These trapping sites are formed when metal complex particles are incorporated into the host polymer. The *E*_g_ values achieved from the relationships between (*αhv*)^1/*γ*^*versus* photon energy for the polyvinyl alcohol composites are presented in Table 3 for the direct and indirect transitions.

**Table 3:** Calculating optical dielectric loss and determining the optical band gap using Tauc's model

Sample designation	*E*_g_ (eV) for *γ* = 1/2	*E*_g_ (eV) for *γ* = 3/2	*E*_g_ (eV) for *γ* = 2	*E*_g_ (eV) for *γ* = 3	*E*_g_ from *ε*_i_ (*hv*) plot
PVZMC0	6.21	5.95	5.85	5.22	6.05
PVZMC1	2.52	2.18	1.84	1.55	2.35
PVZMC2	2.45	1.94	1.74	1.17	2.26
PVZMC3	2.35	1.59	1.62	0.84	1.95
PVZMC4	1.98	1.24	1.35	0.38	1.74

The presence of the Zn-metal complex has an evident effect on the decrease in *E*_g_ values in such a way that clean PVA has wide *E*_g_, while PVA composites, including PVZMC1, PVZMC2, PVZMC3, and PVZMC4, have small *E*_g_ values. The insertion of Zn-metal complexes into PVA composite films is responsible for the observed shift in the energy gap. Several OH, NH, and C=O functional groups around the Zn central metal help to interact with the OH groups of the PVA through hydrogen bonding. This produces a polymer–metal complex interaction and overlaps many orbitals, thus reducing the optical band gap.

The direct incorporation of salt into a polyvinyl alcohol (PVA) polymer solution is called a polymer electrolyte. The results of the current study confirm that the polymer–salt system shows negligible optical band gap reduction (see Fig. 6). The novelty of the present work is that the transfer of metal salts into metal complexes and then their combination with polar polymers is surprising in delivering polymer composites with the desired optical band gap. The effect of metal complexes in PVA (complex polymer) on the energy band gap of polymers can depend on various factors, including the nature of the metal complex, coordination bonds between central metal and ligands and ligand functional groups, but in direct salts in PVA (polymer electrolyte) depend on the type of salt.^36^ Bhargav *et al.*^53^ studied the *E*_g_ values of PVA : NaI electrolytes, and they observed that absorption edges reduced from 5.80 to 4.90 eV. Gupta *et al.*^79,80^ observed that the metal complex (chloropentamminecobalt chloride [Co(NH_3_)_5_Cl]Cl_2_) decreased the energy band gap of PVA from 4.8 to 1.85 eV.

**Fig. 12:**
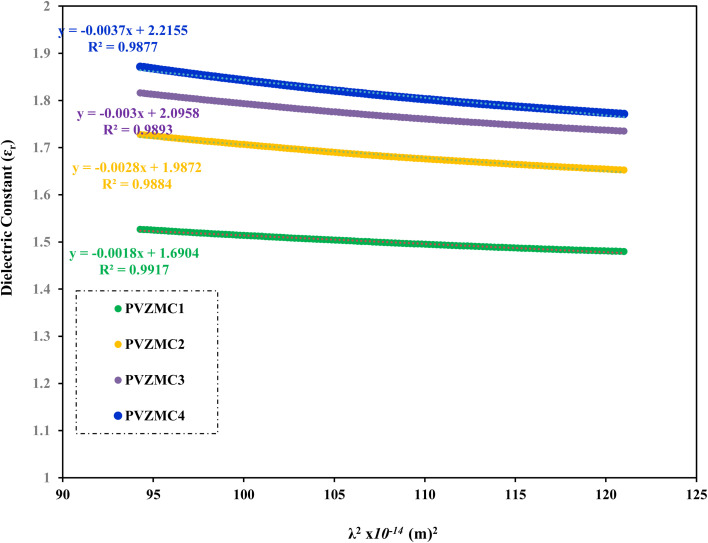
Plot of optical dielectric constant *vs. λ*^2^ of pure PVA and PVA/Zn-metal complex films.

Fig. 12 demonstrates the linearity of the graphs depicting the direct relationship between the real part of the dielectric constant and *λ*^2^ within specific ranges, confirming the validity of the Spiter–Fan equation.8
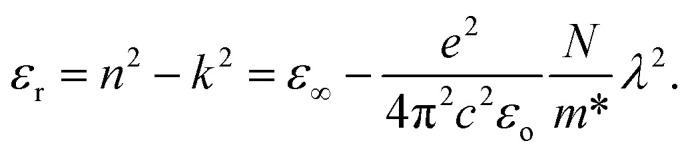


The material's dielectric response at high frequencies within the lattice, particularly at short wavelengths, is represented by the symbol *ε*_∞_. The speed of light (*c*), an electron's charge (*e*), the dielectric constant of the empty space region (*ε*_o_), the concentration of charge carriers (*N*), and the electron effective mass (m*) are all defined in this context, and the constant values are shown in Table 4. The *N*/m* is the ratio of the free concentration of carriers to the effective mass of the electron.^9,81^ The parameters were derived by analyzing the slopes and intercepts of Fig. 12, as depicted in Table 5.

**Table 4:** Different physical quantities used to measure *N*/m* (PVA/Zn-metal complex films)

Physical parameter constants	Values
Electron mass (*m*_e_)	9.109 × 10^−31^ kg1.602 × 10^−19^ C
Electron charge (*e*)	
The dielectric constant of free space (*ε*_o_)	8.85 × 10^−12^ F m^−1^
Speed of light (*c*)	3 × 10^8^ m s^−1^
*Π* (Pi)	3.1415
Effective mass (m*)	10.566 × 10^−31^ kg

**Table 5:** Optical dielectric properties of PVA and PVA/Zn-metal complex films

Sample designation	*N*/m* × 10^55^ m^3^ kg^−1^	*ε*_∞_
PVZMC0	4.87	1.3515
PVZMC1	21.9	1.6901
PVZMC2	34.1	1.9872
PVZMC3	36.5	2.0959
PVZMC4	45	2.2155

The increased density of states within the valence band could be the outcome of adding a Zn-metal complex to the host polar PVA matrix. It is possible to create localized sub-bandgap states by introducing a metal complex. When metal complexes are added, the absorbed edge moves towards lower photon energies.^82^

#### 4.3.3. Urbach energy as a measure of order or disorder

**Fig. 13:**
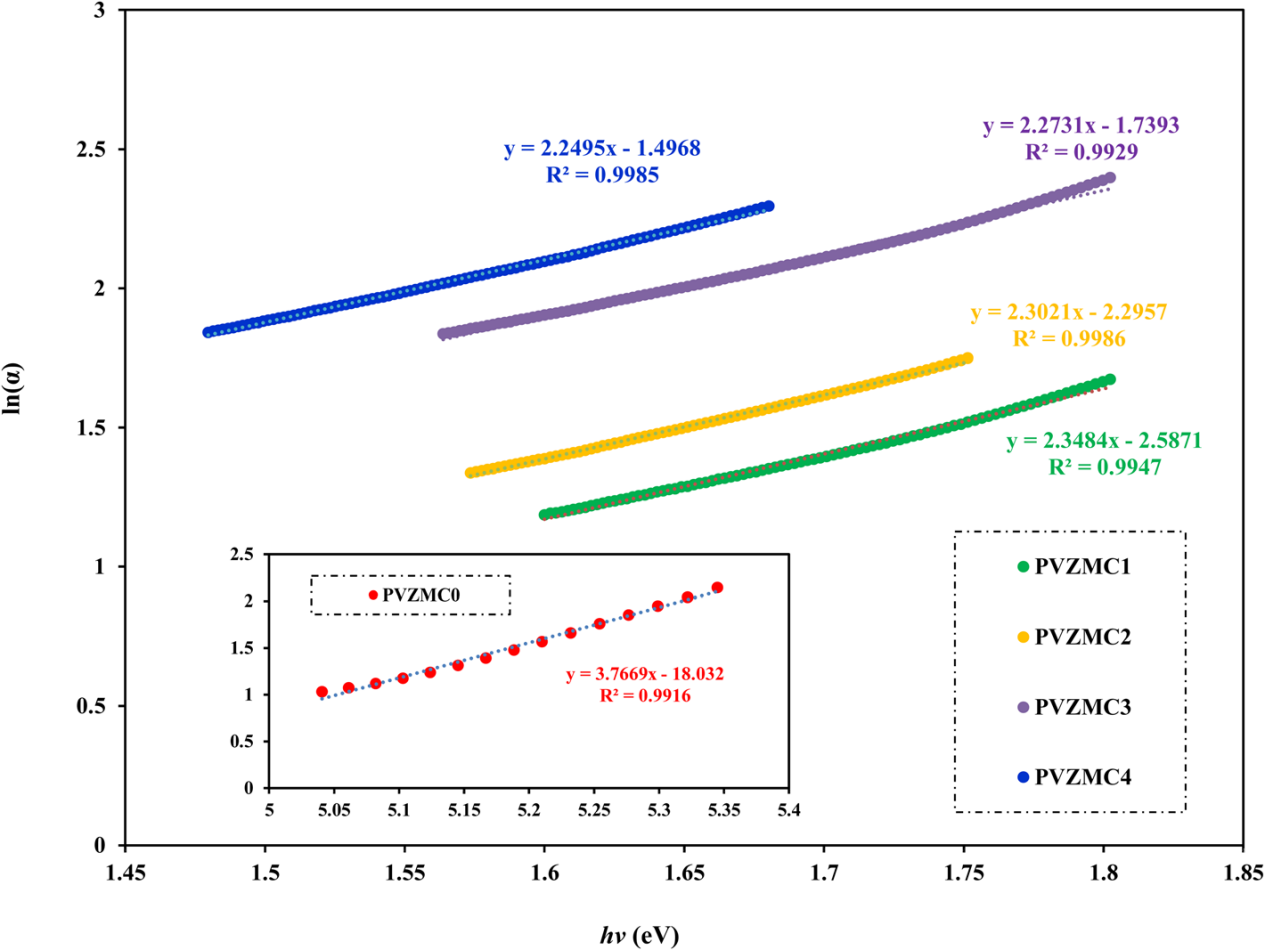
ln(*α*) *versus* photon energy *hv* is used to calculate the Urbach energy for pure PVA and PVA/Zn-metal complex films.

Urbach energy is a parameter that is correlated with absorption coefficient (*α*). Fig. 13 shows the natural logarithm of the absorption coefficient (*α*) as a function of the incident photon energy (*hv*), which is used to perform the Urbach energy calculation. In 1953, Urbach verified the connection between the Urbach energy and absorption coefficient *α*(*v*) at lower absorption levels or longer wavelengths in the spectrum.^83^9
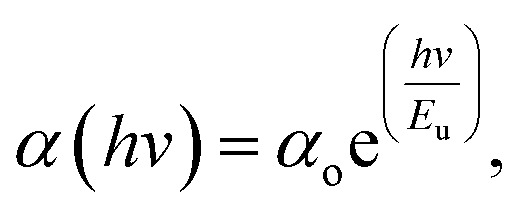
*E*_u_ denotes Urbach energy and *α*_o_ denotes a constant. Apart from the incident photon's energy (*hv*), the optical energy band gap (forbidden gap) is the only factor determining the value of *α*_o_. The Urbach energy (*E*_u_) is a standard tool for measuring the internal disorder of a system, usually derived from the breadth of the forbidden band gap tail of localized states. According to Urbach's rule, each sample exhibits an exponential correlation between the absorption coefficient (*α*) and the photon energy (*hv*). The Urbach energies (*E*_u_) displayed in Table 6 were calculated with the help of Fig. 13. The reciprocal of the slopes of the linear data is equivalent to *E*_u_. The density of localized states grows in proportion to the concentration of the Zn-metal complex. Furthermore, overlapping localized states might magnify the mobility gap caused by higher doping concentrations. The concept of overlapping localized energy states may explain the Urbach Energy (*E*_u_) increment as the concentration of Zn-metal complex doped increases. The energy band gap is reduced as a result of this overlapping effect.^84^

**Table 6:** The present study introduces an empirical single-oscillator model, known as the W–D model, and the Urbach energy of pure PVA and PVA/Zn-metal complex samples for calculating optical bandgap energy

Sample designation	*E*_d_ (eV)	*E*_o_ (eV)	*n*_o_	*E*_u_ (eV)	*E*_g_ (eV)
PVZMC0	1.610106	5.903775	1.128151	0.2654	6.05
PVZMC1	0.819452	2.272914	1.166417	0.4258	2.3
PVZMC2	1.016202	2.152824	1.213273	0.4343	2.1
PVZMC3	1.176863	2.186611	1.240247	0.4399	2.02
PVZMC4	1.12438	2.074594	1.241763	0.4445	1

The values of the *E*_u_ increased from 0.2654 to 0.4445 eV as the Zn-metal complex amount increased from 9 to 36 mL. The increase in *E*_u_ value represents the growing amorphous nature of the PVA/Zn-metal complex matrix, leading to increased atomic and disorder defects in the structural bonding. Disorders and defects can result in localized states occurring at or near the connecting band level. The results of this study are consistent with the XRD results (see Fig. 1).

#### 4.3.4. Wemple–DiDomenico model

The values of the Wemple–DiDomenico model WD parameters, dispersion energy (*E*_d_) and oscillator energy (*E*_o_), along with the static refractive index, *n*_o_, were obtained from the analysis, as shown in eqn (10) at ℏ*ω* → 0 or (*hv* → 0).^85–87^10
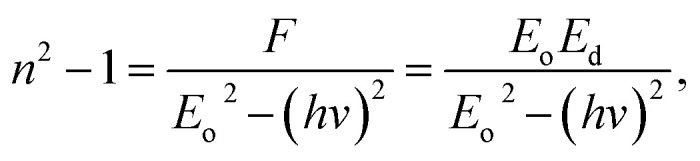
where 
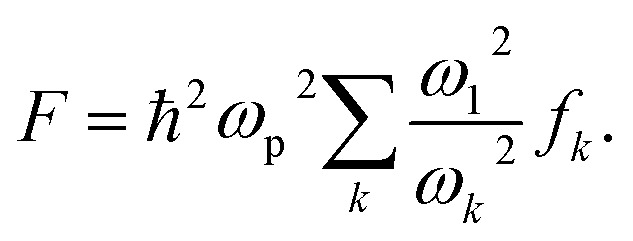


**Fig. 14:**
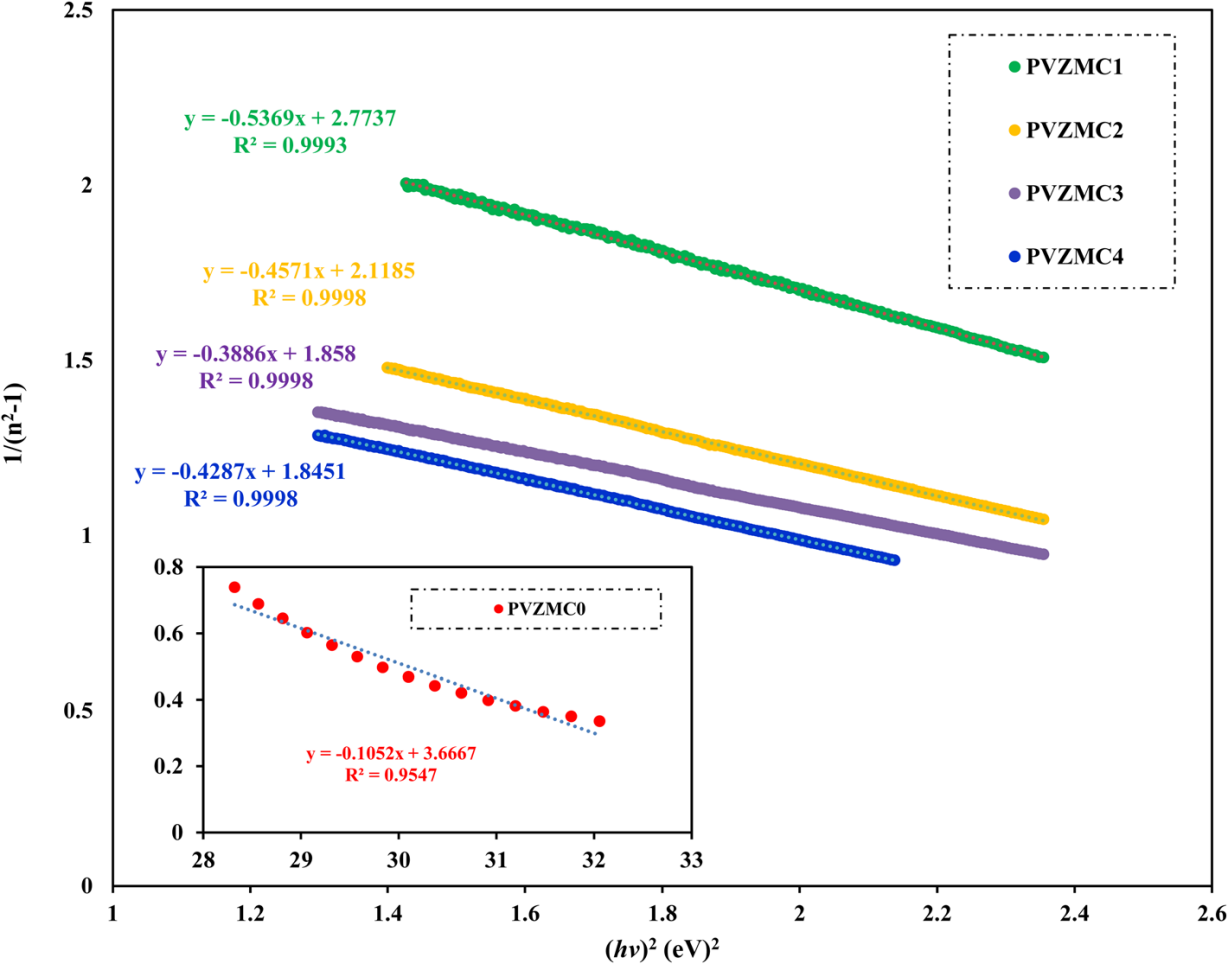
Variant 1/(*n*^2^ – 1) against photon energy (*hv*)^2^ for pure polymer PVA and composite PVA/Zn-metal complex films.

The photon energy is denoted by *hv*. The electric-dipole oscillator strength, denoted by *f*_k_, is linked to transitions occurring at frequency *ω*_p_. The plasma frequency, *ω*_p_, is defined as 
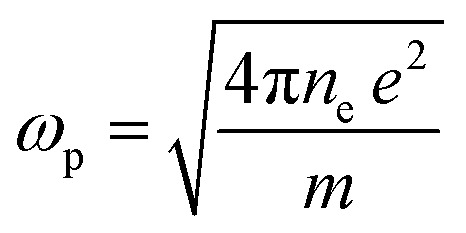
, where *n*_e_ represents the effective electron density, and *e* and *m* represent the charges and mass of the electrons, respectively. According to the Wemple and DiDomenico (WD) hypothesis, one of the oscillators within the spectral range is believed to possess greater strength than the others.^85^Fig. 14 shows the plot of 1/(*n*^2^ − 1) against (*hv*)^2^. The values of *E*_d_ and *E*_o_ were determined from both the slope and the intercept of the regression lines, respectively. The determined values are presented in Table 6. The *E*_d_ and *E*_o_ results exhibit a downward trend with increasing concentrations of Zn-metal complexes. The single effective oscillator energy, denoted as *E*_o_, is close enough to the optical band gap achieved from the Taucs model. This establishes the fact that the data analysis was performed very carefully.

**Fig. 15:**
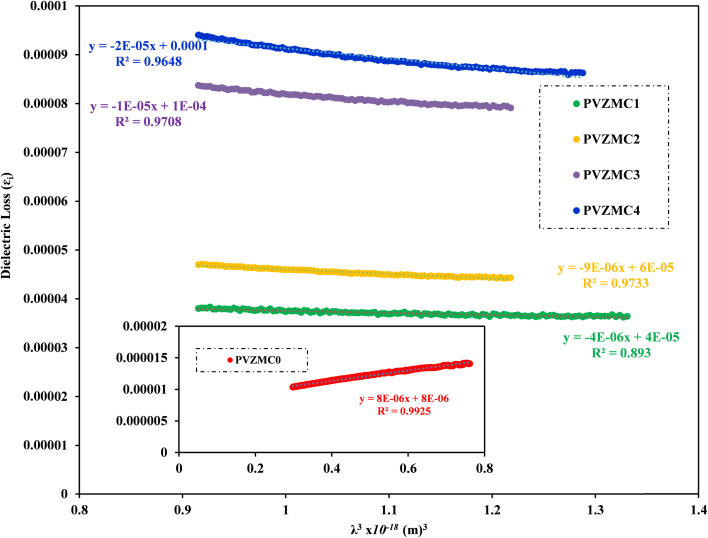
Plots of *ε*_i_*versus λ*^3^ for the pure PVA and PVA/Zn-metal complex films.

In this study, we examine the dielectric loss (*ε*_i_) in relation to the wavelength of the incident photon to ascertain the relaxation time (*τ*).^87^Fig. 15 illustrates the plot of (*ε*_i_) as a function of the cubic of photon wavelength (*λ*^3^) for all films of polyvinyl alcohol (PVA) and PVA/Zn-metal complex composites. The time constant (relaxation time) *τ* can be ascertained by analyzing the gradient of the plot of *ε*_i_ against cubic wavelength *λ*^3^ and by utilizing the calculated value of *N*/m* derived from the following equation:11
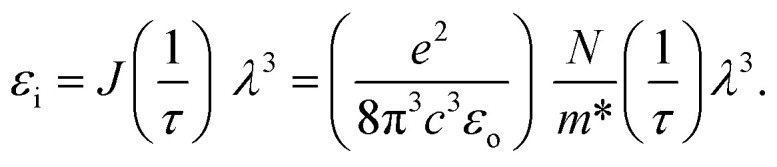


In addition, the optical mobility parameter (*μ*_opt_); optical resistivity, which refers to the measurement of the resistance of a material to the flow of light (*ρ*_opt_); and the plasma frequency *ω*_p_ using the Drude free electron model can be determined by considering the ratio term *N*/m*, the electronic charge *e*, and the electric permittivity of air *ε*_o_.^87^ The calculated values of optical mobility refer to the ability of particles or molecules to move under the influence of light (*μ*_opt_) and optical resistivity (*ρ*_opt_), and the frequency of plasma *ω*_p_ is illustrated in Table 7.12
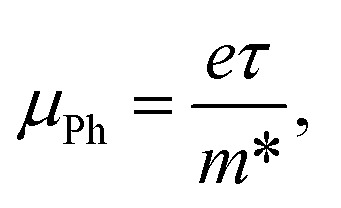
13
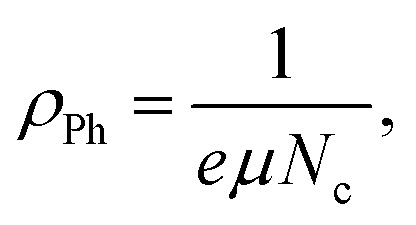
14
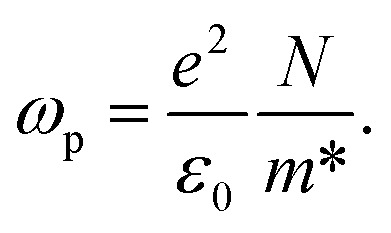


**Table 7:** Determined values of *τ*, *μ*_opt_, *ρ*_opt_, *N*_c_ and *ω*_p_ of pure PVA and PVA/Zn-metal complex

Sample designation	*τ* × 10^−12^ s	*μ*_opt_	*ρ*_opt_ × 10^−8^ Ohm m	*N*_c_ × 10^25^	*ω*_p_ × 10^29^ Hz
PVZMC0	2.6642	0.4	31.5772	4.89	1.4107
PVZMC1	20.3978	3.64	0.7797	20.20	6.3482
PVZMC2	10.6577	2.51	0.7250	30.43	9.8749
PVZMC3	10.5985	2.42	0.7017	30.67	10.5803
PVZMC4	0.9858	1.49	0.9226	40.53	13.0490

The light beam is slowed down through sheets of polymer composite with substantially greater refractive indices, leading to the low values of *τ*, *μ*_opt_, and *ρ*_opt_. After adding the Zn-metal complex, the electron's *w*_p_ jumped from 1.41 × 10^29^ Hz to 1.304 × 10^30^ Hz. The plasma frequency similarly increases when the Zn-metal complex increases, which can be attributed to the increase in carrier concentration *N*, assuming that the effective mass m* remains constant.^5,6^ The quantitative measurements of these parameters for polymer composites are crucial for recognizing their suitability in optical devices.^88^

### 4.4. Non-linear optical (NLO) properties

Nonlinear optics is a modern and adaptable scientific area that explains how light interacts with matter by showing how induced polarization changes nonlinearly in response to external electric and magnetic fields. Nonlinear optical (NLO) phenomena occur when a substance emits electromagnetic radiation with different amplitudes, phases, and frequencies. Medium polarization (*P*) occurs when valence electrons transfer charges to atoms in the medium due to the induced electric field as light travels through the medium. The equation shows that an electric field's strength is directly proportional to the degree of polarization in a given medium.^89^

15 *p* = *ε*_o_*χ*^(1)^*E* + *ε*_o_*χ*^(2)^*E*^2^ + *ε*_o_*χ*^(3)^*E*^3^ +…

**Fig. 16:**
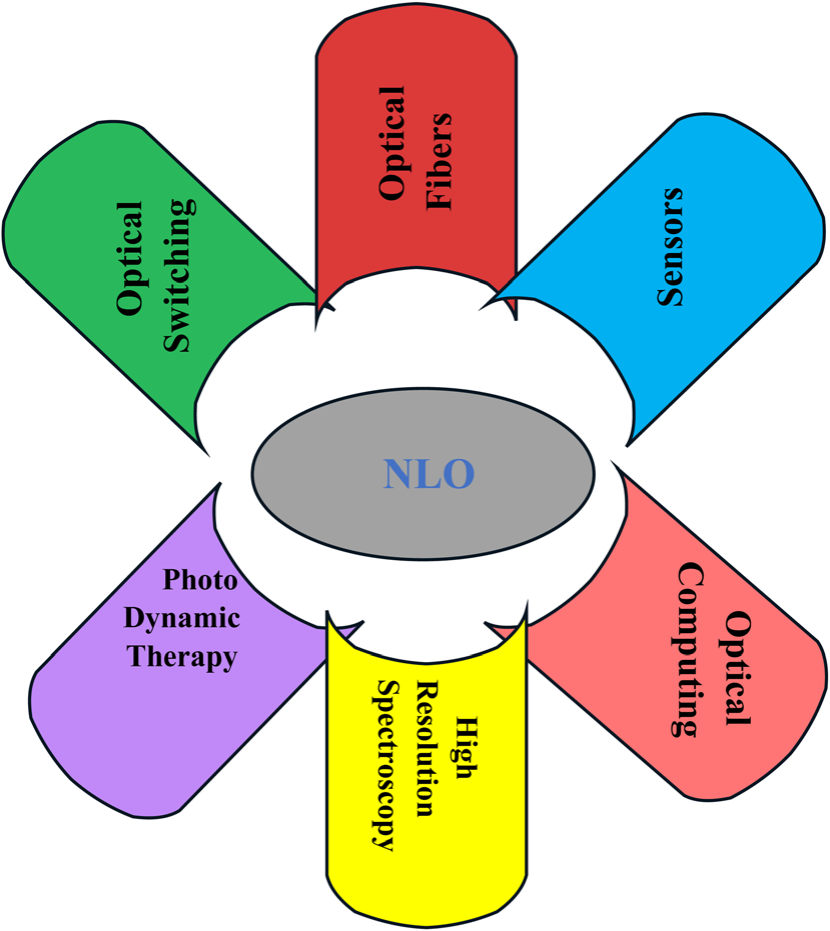
Nonlinear optical materials in various application fields.

Vacuum dielectric constant (*ε*_o_), applied electric field strength (*E*), and macroscopic polarization strength (*P*) are all used in the preview equation; *χ*^(1)^, *χ*^(2)^ and *χ*^(3)^ are the first, second and third-order linear susceptibilities that refer to the measure of the response of a material to an applied electric field of EM radiation, which is directly proportional to the field strength.^90^Fig. 16 shows the application of the nonlinear optics (NLO). From the figure, it can be understood that the NLO has impacted the development of many fields of science that are too crucial to be emphasized.^91^

**Fig. 17:**
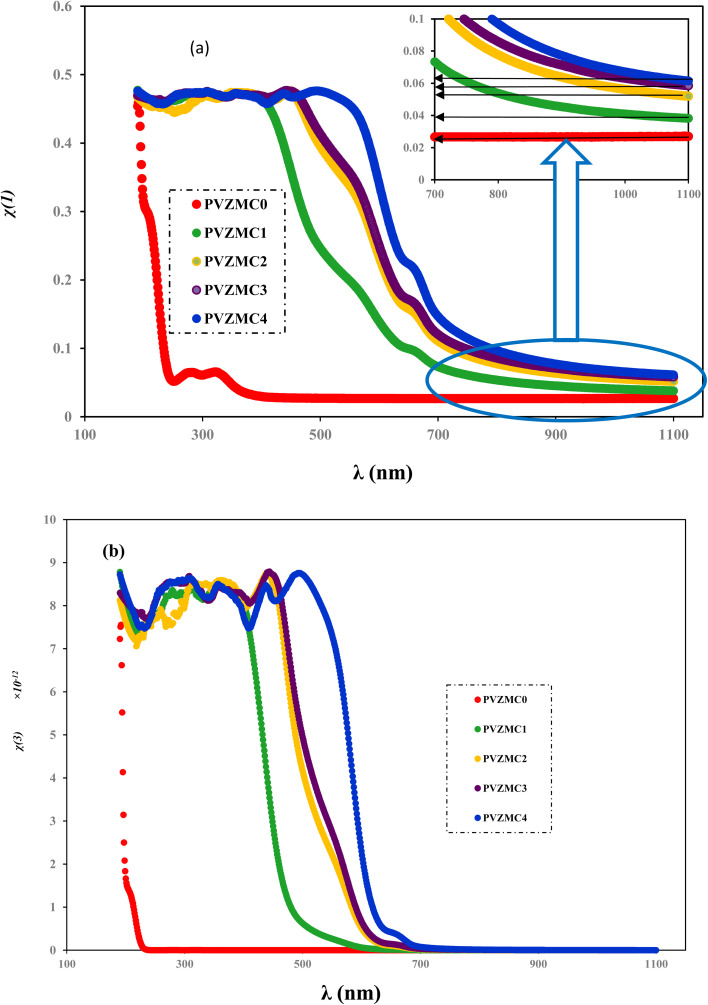
Linear and non-linear plots of (a) *χ*^(1)^ and (b) *χ*^(3)^*vs. λ* for the pure PVA and PVA/Zn-metal complex films.

Doping PVA with Zn-metal complex techniques can significantly improve the optical linear and non-linear characteristics of these composites. The variation in the band gap resulting from doping is caused by the creation of defect states within the band gap, which leads to a drop in the band gap or the elimination of inherent defect states in the samples, resulting in a decrease in the band gap.^92^ By analyzing the graphs, we can ascertain the values of *χ*^(1)^ (linear optical susceptibility) and *χ*^(3)^ (third-order nonlinear optical susceptibility) by drawing a straight line from the plateau region to the *y*-axis, as shown in Fig. 17. The values of *χ*^(1)^ are illustrated in Table 8. The linear optical parameters *χ*^(1)^ were also determined using the Wemple and DiDomenico (W–D) model for a PVA system doped with metal complexes. The increase in the value of *χ*^(1)^ is correlated with the presence of transition metals in the PVA polymer matrix. This phenomenon is associated with the dependence of the refractive index on the energy of the incident photons.^91^16
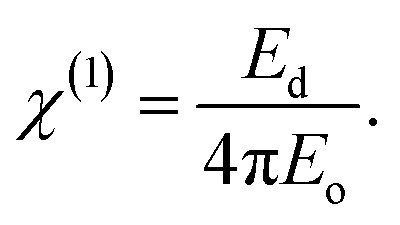


**Table 8:** Non-linear optical parameters of pure PVA and PVA/Zn-metal complex films

System designation	*χ* ^(1)^ (graph)	*χ* ^(1)^ (W–D)	*n*_NL_ × 10^−12^
PVZMC0	0.025	0.0217	0.011
PVZMC1	0.043	0.0286	0.07
PVZMC2	0.050	0.0375	0.125
PVZMC3	0.055	0.0428	0.17
PVZMC4	0.061	0.0431	0.185

**Fig. 18:**
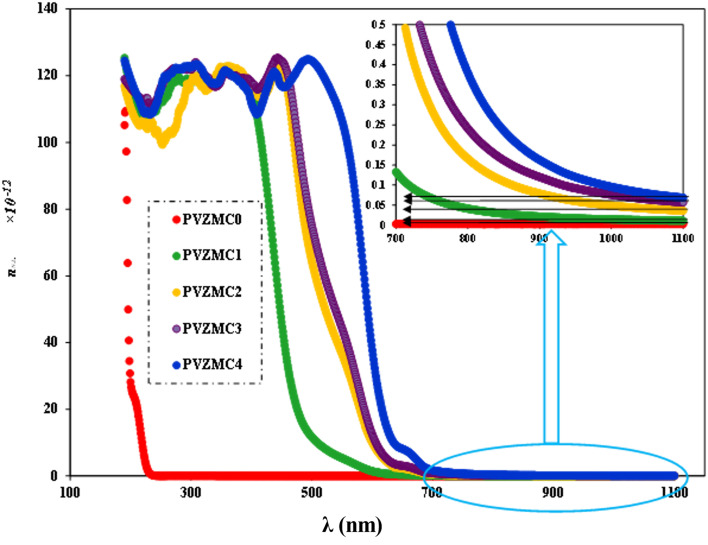
Nonlinear refractive index *n*_NL_*versus λ* for PVA/Zn-metal complex films.

The values of the linear parameter *χ*^(1)^, the nonlinear parameter *χ*^(3)^ and *n*_NL_ are presented in Table 8. Higher values of *χ*^(1)^ are observed for the PVA/Zn-metal complex, as depicted in Fig. 17a. Increased Zn-metal concentrations result in more particles that absorb electromagnetic waves, thus enhancing the polarization of the polymer sheets and improving the nonlinear characteristics. As the energy gap narrowed, the nonlinear characteristics of the polymeric composites may improve. The values of optical susceptibilities and *n*_NL_ of composites are greater than those of pure PVA. Hence, the hybrid films could be utilized in nonlinear optoelectronic devices. It is interesting to mention that the higher values of *n*_NL_ and *χ*^(3)^ indicate the suitability of PVA : Zn-metal complex composites for utilization in nonlinear optical systems.^93^Fig. 18 shows the relationship between the non-linear refractive index and photon energy.

We employed two parameters to characterize the optical transitions of electrons in the PVA/Zn-metal complex composite films: the surface energy loss function (SELF) and the volume energy loss function (VELF), which show an electron transition at high and low wavelengths in bulk materials. The SELF and VELF are of high interest because they are related to microscopic phenomena inside the materials, and they can be deduced from optical dielectric parameters. The values of the SELF and VELF were determined utilizing detailed relationships with the *ε*_r_ and *ε*_i_ components of the dielectric constants:17
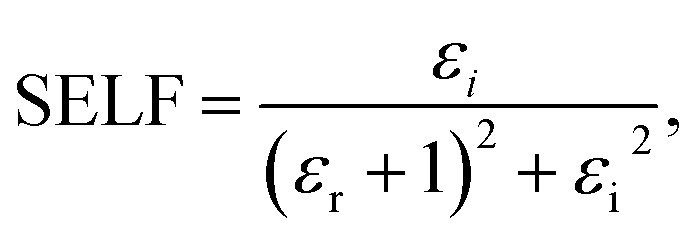
18
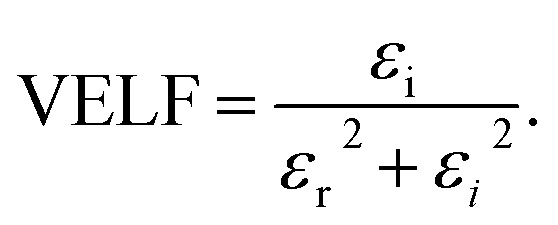


**Fig. 19:**
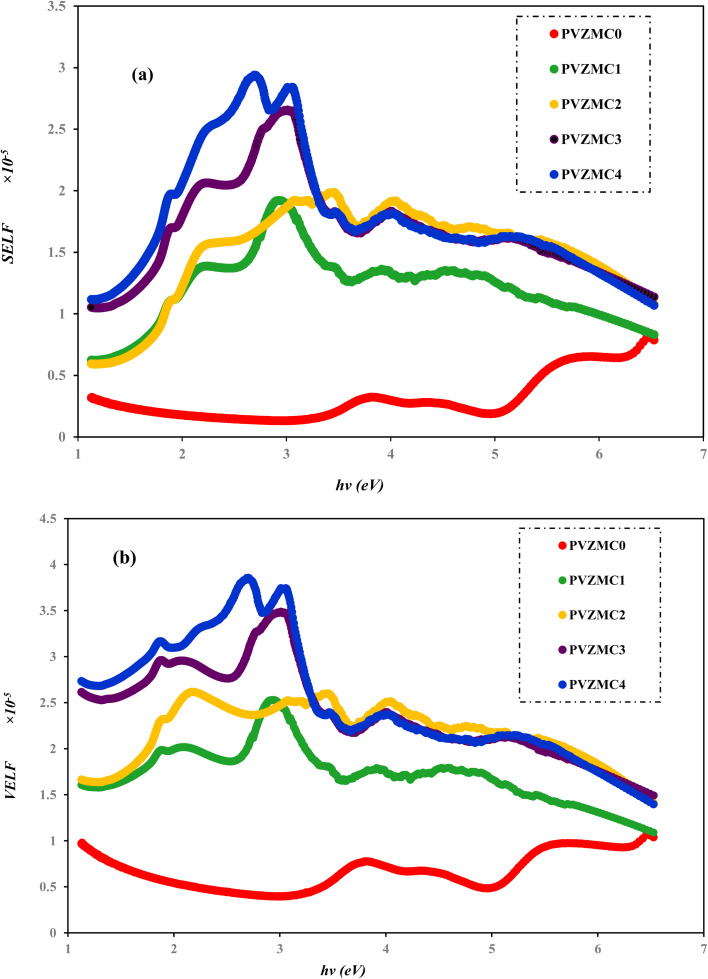
(a) SELF and (b) VELF *vs.* photon energy functions for the PVA and PVA/Zn-metal complex films.

Fig. 19 shows that the SELF and VELF are altered due to changes in Zn-metal complex concentrations, resulting in a modification of electron transition energy. The increase in SELF and VELF signifies a reduction in vacant energy levels produced within the host band gap of the PVA. Thus, the presence of many new energy states assigned to the added Zn-metal complex increases the probability of light–matter interaction. The VELF function values had greater magnitudes than the SELF values, as shown in Fig. 19, because the bulk contains more electrons than the surface. This demonstrated that doping can alter the transition energy of electrons.^94^

## 5. Conclusions

The main findings of this study are that polymer composites based on green-synthesized metal complexes are guaranteed to reduce the optical band gap. Dyes from green tea are sufficient to capture dangerous heavy metal ions and transfer them to safe precipitated solid metal complexes. The FTIR results establish that Zn metal is surrounded by numerous functional groups that are suitable for complexation with polar groups of polar polymers. The XRD and FTIR outcomes confirm strong interaction among functional groups of the polymer and Zn-metal complex particles. The primary finding of this study is the production of amorphous polymer composites that closely align with the energy band gap of semiconductors. These composites have significant relevance for use in optoelectronic devices. The X-ray diffraction results and the Urbach energy parameter demonstrate the transition of the semicrystalline phase of PVA to an amorphous phase. The values of the *E*_u_ increased from 0.2654 to 0.4445 eV as the Zn-metal complex amount increased from 9 to 36 mL. The addition of metal complexes caused a shift in the dispersion behavior of the refractive index of PVA towards longer wavelengths. The refractive index, the static refractive index and non-linear refractive index increase from (1.1553 to 1.3569), (1.128 to 1.2417) and (0.011 to 0.185) by the incorporation of PVA with the metal complex. The Tauc model was employed to determine the most likely type of electron transition. Increased density of states *N*/m* within the valence band could result from adding a Zn-metal complex to the host PVA matrix. The dispersion energy, third-order optical susceptibility and nonlinear refractive index increased with the increase in Zn-metal complex concentration. The increase in SELF and VELF signifies that new energy states assigned to the added Zn-metal complex increase the probability of light–matter interaction; thus, more losses were expected in doped composite films. Due to advancements in enhanced linear and nonlinear optical characteristics, PVA composite films can be utilized in optical and optoelectronic devices.

## Data availability

Data will be available from the corresponding author upon request.

## Author contributions

Dana S. Muhammad: methodology, investigation, analysis, validation, writing – original draft. Dara M. Aziz: validation, supervision, project administration, writing – review & editing. Shujahadeen Aziz: conceptualization, writing – review & editing, supervision, project administration.

## Conflicts of interest

The authors declare no conflict of interest.
